# Experimental production of K-rich metasomes through sediment recycling at the slab-mantle interface in the fore-arc

**DOI:** 10.1038/s41598-023-46367-7

**Published:** 2023-11-10

**Authors:** Fatma Gülmez, Dejan Prelević, Michael W. Förster, Stephan Buhre, Jennifer Günther

**Affiliations:** 1 Department of Geological Engineering, Istanbul Technical University, 34469 Istanbul, Türkiye; 2 Faculty of Mining and Geology, University of Belgrade, Đušina 7, 11000 Belgrade, Serbia; 3 Institut fur Geowissenschaften, Johannes-Gutenberg-Universität, 55099 Mainz, Germany; 4 School of Natural Sciences, Macquarie University, Sydney, NSW 2109 Australia

**Keywords:** Solid Earth sciences, Petrology

## Abstract

Sediment contribution to the mantle is the key step for the generation of orogenic magmatism to produce its isotopic and geochemical inventory. Even though they are exceptional for the post-collisional settings, there are worldwide examples of arc-related ultrapotassic mafic magmas which require complex multi-stage processes along with sediment melting e.g. in Italy or Pontides of Türkiye. To understand the metasomatism leading mantle to produce ultrapotassic mafic melts, we simulated the reactions of depleted (harzburgite) and fertile (lherzolite) mantle with subducted carbonate-rich sediment at relatively cold (800–850 °C) and shallow (2 GPa, 60–80 km) slab-mantle interfaces. The melting of sediments can trigger the formation of immiscible and conjugate carbonatitic and silicic melts which flux the mantle to develop hydrous minerals and dolomitic melt. The metasomatic growth product is a wehrlite composed of clinopyroxene, phlogopite, carbonate minerals and amphibole, representing a source of choice for Si-undersaturated ultrapotassic lavas. The occurrence of conjugate carbonatitic and silicic melts and their potential physical separation, offer a possibility for fractionation of several canonical trace element ratios such as Th/La, observed in Si-saturated ultrapotassic lavas. The synergy between peridotite-melt interaction and the physical separation of the carbonatitic and extremely K-enriched silicic melts are essential for the compositional evolution of ultrapotassic orogenic magmas and their mantle sources.

## Introduction

Sediment recycling within the mantle wedge contributes considerably to the geochemistry of arc magmas^1^. In orogens like the Alpine-Himalayan orogenic belt (AHOB), this recycling is not only essential for the composition of the arc volcanism, but also forms metasomatic domains within the lithospheric mantle, so-called metasomes. These metasomes remain stored and activated following the cessation of active-margin processes^2,3^. Carbonates and silicates are major constituents of the sediment load of the subducting column, and their devolatilization and melting represent a principal source of fluids and/or melts that will metasomatize the overriding mantle^4–7^. The mantle will interact with hydrous silicate melts, aqueous and supercritical alkaline siliceous fluids, as well as carbonatitic melts, resulting in the formation of metasomes^8–11^.

The metasomatic effects of the recycling of terrigenous clastic and carbonate sediments on the mantle-wedge are distinct: recycling of siliciclastic sediments will result in enrichment in silica and potassium with depletion in HFSEs relative to LILE whereas it is expected that the carbonate-rich sediments will lead to extreme silica depletion with unusual enrichments in REE evident by geochemical features of the carbonatites (e.g.^12–16^). Available data suggest that the melting of carbonate-rich siliciclastic sediments forms a melt of granitic composition at lower pressures (2.5 GPa) and of phonolitic composition at higher pressures (5.0 GPa)^11^. The formation of Ca-carbonatite, on the other hand, occurs at higher temperatures (> 1100 °C) and pressures ranging from 3.7 to 5.0 GPa.

**Figure 1:**
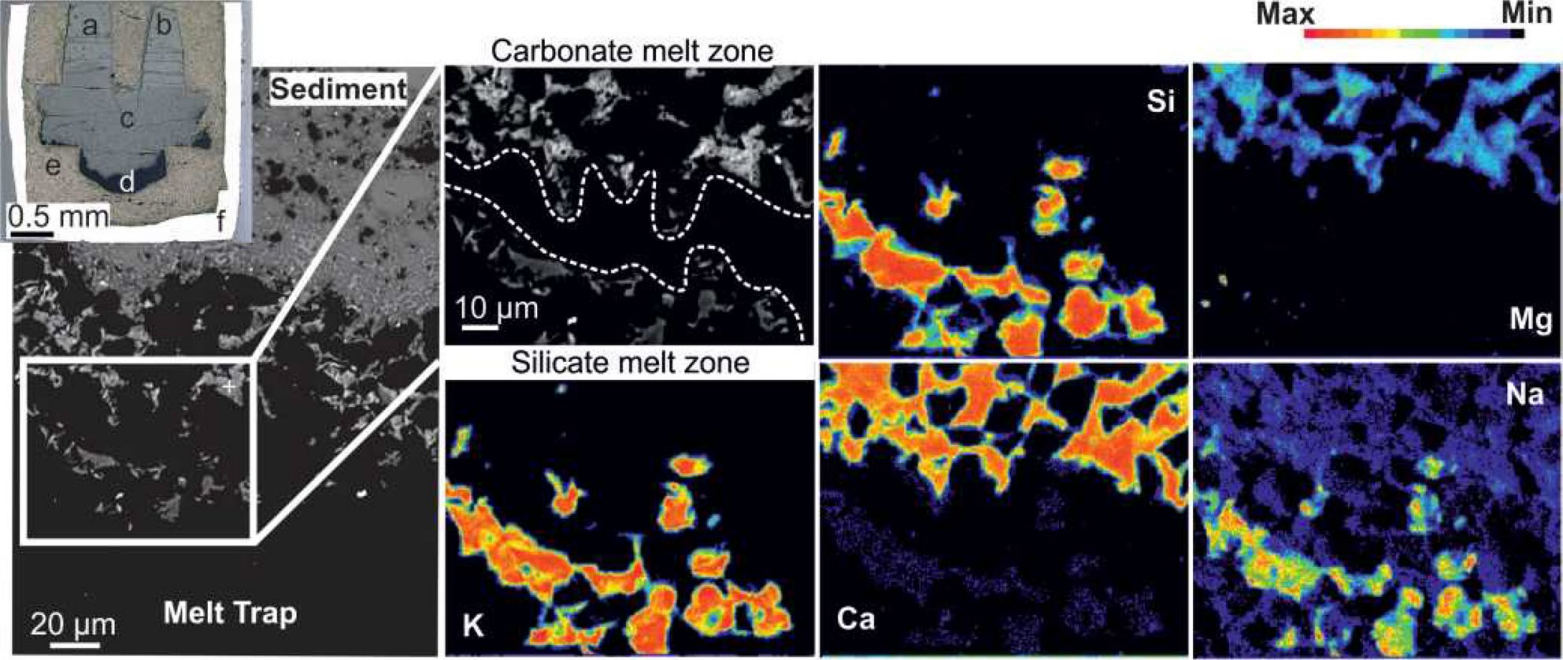
Backscattered electron (BSE) image (left) and elementary maps of stratified melts in the melt trap. In the inset, a photograph (reflected light) of an epoxy-embedded, polished modular capsule is shown with the labels for the capsule components a: harzburgite, b: lherzolite, c: carbonaceous pelite, d: melt trap- diamond grains, e: graphitic inner capsule, d: Au–Pd outer capsule. Elementary mapping (using EPMA) reveals the distribution of Na, Mg, K, Ca and Si highlighting compositional disparities between carbonatitic and silicic melts stemming from wet sediment melting during the reaction experiment at 800 °C/2 GPa. Carbonatitic glass displays elevated Ca and Mg levels while silicic glass shows enrichment in K, Na and Si (Experiment 15B; diamond grains create the black background).

**Figure 2:**
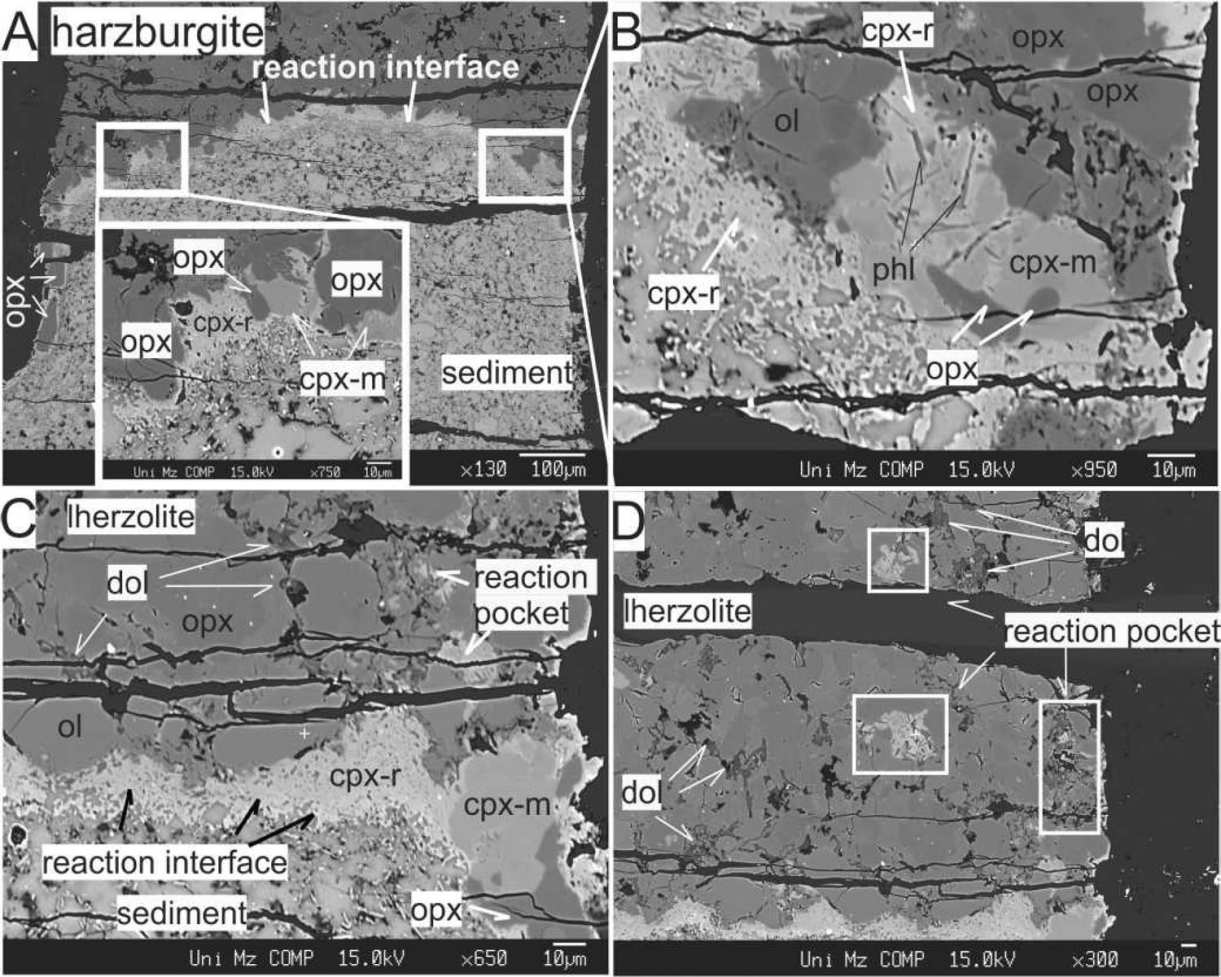
BSE images depicting experimental charges and the emergence of metasomatic minerals resulting from sediment-peridotite reactions. (**A**) The reaction interface seen as a bright film layer between crust and mantle portions in the reaction experiment between sediment and harzburgite at 850 °C/2 GPa, (**B**) Closer view revealing the embayment of Opx by metasomatic Cpx (Cpx-m), the development of sieve texture in Opx and of inclusions of phlogopite needles in Cpx (Cpx-r); olivine (Ol) remained intact, (**C**) The reaction interface dominated by the second generation clinopyroxenes (Cpx-r) and poorly developed reaction pockets seen at right top corner (reaction experiment between sediment and lherzolite at 850 °C/2 GPa), (**D**) Presence of dolomitic melt ponds and reaction pockets (*ol* olivine, *opx* orthopyroxene, *opx-r* second generation orthopyroxene, *cpx-m* metasomatic clinopyroxene, *cpx-r* second generation clinopyroxene, *phl* phlogopite, *cb* carbonate minerals, *amp* amphibole, *dol* dolomitic glass).

**Figure 3:**
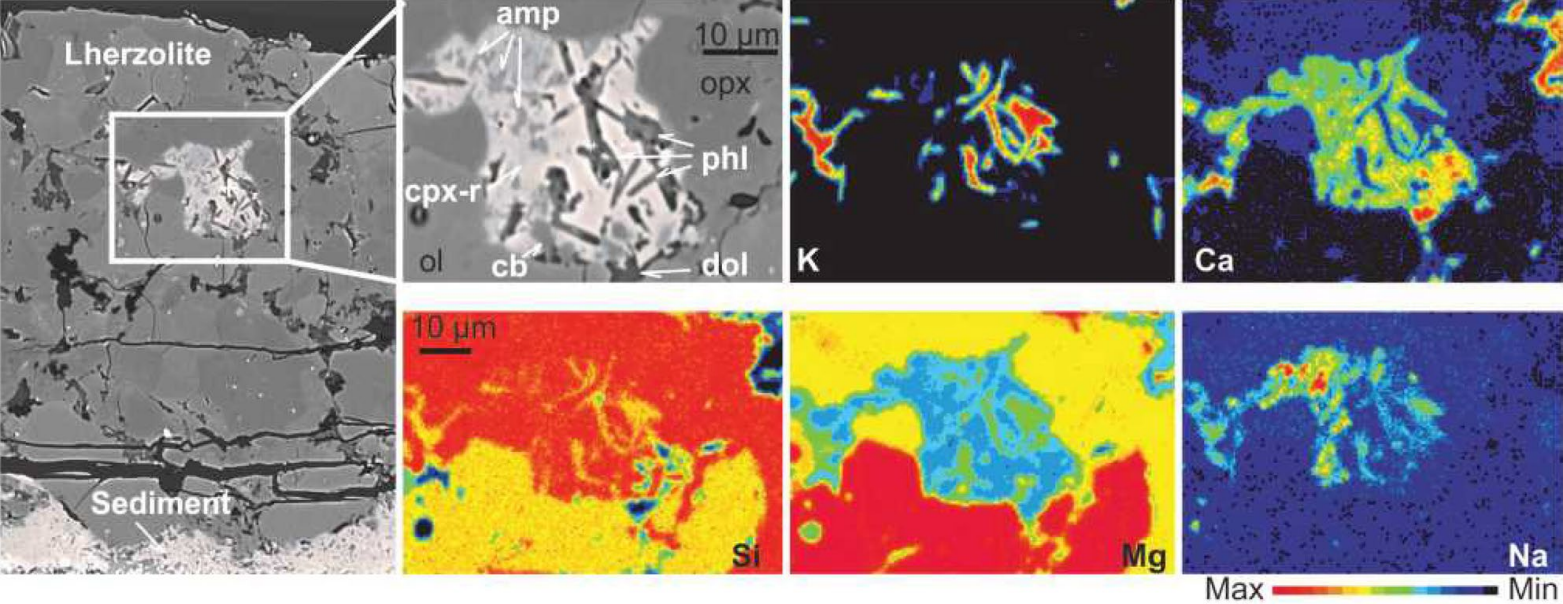
BSE images (upper left side) and elementary maps of the metasome formed as a result of the reaction between carbonaceous pelite and lherzolite at 850 °C/2 GPa within the peridotite portion. The distribution of K, Ca, Si, Mg and Na reveals the possible mineral paragenesis of the metasome (Experiment E10) (see Fig. 2 caption for the abbreviations).

In this study, we have performed a series of reaction experiments between sediment and peridotite in a piston-cylinder apparatus at 800 and 850 °C and 2 GPa, and water-rich conditions. Natural carbonaceous pelite was combined with synthetic harzburgite and lherzolite, either co-loaded in modular experimental capsules containing both peridotite types or individually in simple experimental capsules (see "Supplementary Data File I"). The conditions mimic scenarios where the strong dehydration of serpentinized peridotites beneath the oceanic crust triggers flux melting of the overlaying carbonaceous pelites. Our experiments suggest that carbonate-rich siliciclastic sediments have the potential to produce conjugate carbonatitic and silicic melts (Fig. 1) that form metasomatic domains within harzburgitic and lherzolitic mantle (Fig. 2). Importantly, the produced metasomes consist of clinopyroxene + phlogopite ± amphibole ± carbonate minerals (Fig. 3) that would be capable of producing Si-undersaturated ultrapotassic melts (e.g. leucitites and kamafugites) during the further stages of orogenesis that activate the accreted fore-arc mantle lithosphere.

## Fore-arc mantle as a metasome reservoir

In orogenic belts like AHOB, a significant portion of Tertiary volcanic associations is characterized by universal enrichment of potassium coupled with invariably high incompatible trace element contents and an isotopic signature that compositionally overlaps the upper continental crust^4–6,17–21^. This has for a long time been a puzzling issue, especially for the mantle-derived lamproites (Si-saturated), and leucitites and kamafugites (Si-undersaturated), which have the most extreme compositions: K_2_O up to 12 wt%, ^87^Sr/^86^Sr up to 0.723, εNd down to − 13 as well as highly forsteritic olivine (For up to 93%) with δ^18^O_V-SMOW_ values up to + 11.5‰^3,4^. These data establish a connection between the volcanism and massive crustal recycling that must have played a crucial role in the mantle source preconditioning of AHOB^5,6^, and represent a global feature^17–19^. However, it is still enigmatic which segment of the arc architecture might constitute this enrichment and what physical and chemical mechanisms might underlie it. Several recent studies point to the fore-arc mantle as a potential site for this recycling, with some of the best-documented case studies coming from AHOB, including the Upper Cretaceous Pontide and South Aegean arcs, but also Indonesian Batu Tara, Trans Mexican Volcanic Belt, Central and Southern Vosges Mts. of northeastern France within the European Variscan Belt etc^22–27^. A few scenarios concerning the physical mechanism of the fore-arc mantle preconditioning have been in previous literature. The involvement of small continental slivers that are characteristic of accretionary orogens such as those produced by the Cenozoic closure of the Tethys Ocean in the Alpine-Himalaya belt or modern-day Indonesia is of profound significance for this recycling^5,22,26,28^. In contrast to the steady-state subduction zone, their presence will ultimately provide isotopically old crustal material reappearing later in post-orogenic lavas^3^. An alternative mechanism to arrive at a similar result is the stacking of mixed “cold plumes” under the orogenic wedge, composed of similar tectonic mélanges but derived from deeper within the subduction channels^29–31^. This model proposes that subducted sediments may move across the Benioff zone due to “delamination” and rise into the overlying mantle wedge^31^ especially when they are carbonate-rich^32^. These "cold plumes" can transport the fertile subducted crustal materials towards hotter zones of the mantle wedge above the subducting plate.

**Table 1:** Experiment type, water source, mantle/crust ratio, capsule design, starting materials, duration and conditions for the experiments.

#		% water	Water source	Mantle/crust	Capsule design	Mantle	Crust	Duration days	T °C	P GPa
E10	Reaction	20	H_2_O	1/3	Modular	AVX & KLB	SD48	14	850	2
E15	Reaction	20	H_2_O	1/3	Modular	AVX & KLB	SD48	14	800	2
E17	Reaction	20	Mg(OH)2	3/5	Simple	AVX	SD48	13	800	2
E25	Reaction	20	Mg(OH)2	3/5	Simple	KLB	SD48	13	800	2
E33	Reaction	20	Mg(OH)2	3/5	Simple	AVX	SD48	13	850	2
E41	Reaction	20	Mg(OH)2	3/5	Simple	KLB	SD48	14	850	2
E45A	Only-sediment	20	H_2_O		Simple		SD48	6	850	2
E45B	Only-sediment	20	H_2_O		Simple		SD48	7	800	2

Six reaction experiments between sediment and peridotite, and two only-sediment experiments were conducted. A natural carbonaceous pelite sample (marlstone, sample no: SD48 in Table 2 of the previous study^6^) was combined with synthetic harzburgite (AVX^33^) and lherzolite (KLB^34^) within two distinct capsule designs as modular and simple (See Supplementary Data File I).

Despite the challenge of providing the exact mechanism of fore-arc preconditioning, existing thermal, geophysical and geochemical models suggest that the area between fore-arc serpentinites and the source region of arc magmas may represent a more efficient ground for sediment recycling than previously assumed^35,36^. Subduction zone seismicity data coupled with new high P–T experiments suggest that in the fore-arc region increased fluid pressures produced by the melting of sediments will promote the occurrence of small magnitude earthquakes and episodic tremor and slip^37,38^. In this region, the melting of the subducted sedimentary material will take place well below 1000 °C, and will be additionally forced by the fluids expelled from the underlying serpentinites^39^. Recent data on the subduction zone slab top temperatures as well as empirically obtained thermal models show that in many subduction zones, hydrous sediments will begin to melt already in the fore-arc region^36,40^. Sediment melting will produce melts of different compositions, depending on the ratio between carbonate and silicate components. In each case, it is expected that the sediment-recycling will not be able to induce melting of the overlying mantle, but will infiltrate and react with peridotite and metasomatize it, producing domains enriched in hydrous minerals^5,22,27^.

## Results

We have carried out eight experiments at temperatures of 800 and 850 °C and a pressure of 2 GPa (Table 1). Two of them are only-sediment experiments, in which the capsule was loaded with the starting material comprised of carbonaceous pelite, whereas other experiments of this study are reaction- experiments in which a layer of the carbonaceous pelite was placed in the bottom of the noble metal capsule and a layer of either lherzolite or harzburgite (or both of them) was placed above the sediment (Fig. 1, inset). In all experiments, a melt trap comprised of fine-grained (10 µm) diamond particles under the sediment layer was placed to capture initial melts/fluids from the melting of sediments in both capsule designs. That means that the melts collected in the diamond trap should most closely represent the partial melts of the sediment, and not the melt reacted with the peridotite. Detailed information on the starting materials and two types of capsule designs are shown in the electronic appendix (Supplementary Data File I). Additionally, comprehensive information about the glass and mineral compositions including the metamorphism of carbonaceous pelite as well as the recalculations regarding mass balance and iron-loss, are presented in the same electronic appendix. The complete dataset containing major and trace element compositions of minerals and glasses can be found in Supplementary Data File II.

### Melt composition

In all experiments, two glass compositions were obtained in the melt trap: carbonate-rich and silica-rich ones which we hereafter interpret as crystallized conjugate immiscible carbonatitic and silicic melts. At fore-arc depths, fluids with low solute concentration are referred to as aqueous fluids while those with high silica concentration (> 65 wt%) as melts^41,42^. Despite the ongoing debates surrounding the conditions influencing the formation and composition of these two liquids, the coexistence of aqueous fluids and melts has been observed in the shallow depths of the mantle wedge at 2 GPa and temperatures ranging from 748 to 926 °C^41,43^. Given the compositional criteria and the conditions under which hydrous silicic melts and aqueous fluids could coexist in our experiments, we define produced liquids as hydrous carbonatitic and silicic melts. Due to the fine-grained diamond grains in the melt trap, achieving a highly polished surface is challenging. Therefore, it decreases the measurement quality and makes it difficult to obtain BSE (backscattered electron) images of a satisfactory quality that could systematically illustrate the mutual relationship between silicic and carbonatitic melts, and potentially offer morphological evidence for the immiscible behaviour. The conjugate melts are in most cases found to be either mingled or segregated within the voids of diamond grains to various extents (Fig. 1). This relationship is a reminiscence of carbonate droplets separated from silicate glass along the veins of olivines observed in metasomatized mantle lherzolites as previously reported^44^. The experiment performed at 800 °C/2 GPa (E15B), revealed that the conjugate melts are stratified (Fig. 1), which was previously suggested to have resulted from density differences between compositionally contrasting melts^45^. Besides two immiscible melts observed in the diamond trap, we have also found the carbonatitic melt in the peridotite part of the capsules regardless of the peridotite composition (Fig. 2B,D).

**Figure 4:**
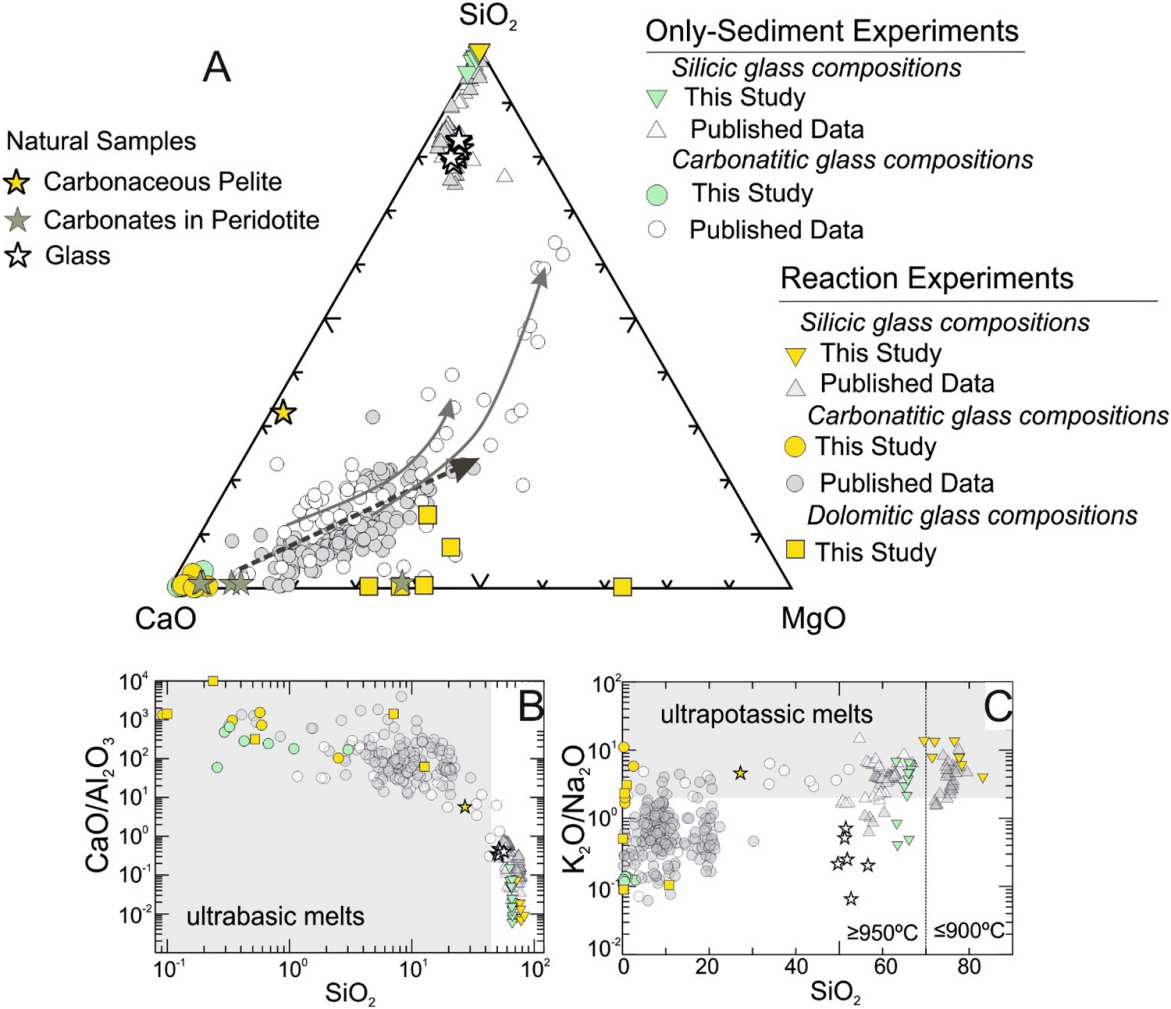
The major element compositions of carbonatitic and silicic glasses in the experiments. Melt compositions obtained from previous experimental studies^11,32,47–50^ and investigations of natural glasses^44,51^. Carbonaceous pelite sample^6^ is shown for comparison. (**A**) Glass compositions shown on SiO_2_—CaO—MgO ternary system, the arrows represent the evolutionary trends with increasing temperature^32^, (**B**) Carbonatitic glasses plot within the ultrabasic melt composition field on SiO_2_ vs. CaO/Al_2_O_3_ diagram, (**C**) Silicic glasses in the experiments represent ultrapotassic melts revealed by on SiO_2_ vs. K_2_O/NaO_2._

The carbonatitic melts have totals as low as 50 wt% and elevated CaO (13.5–48.3 wt%), MgO (0.6–36.5 wt%) and FeO_tot_ (0.7–4.5 wt%) with low NaO_2_ and K_2_O (av. 0.4 wt% and 0.08 wt%, respectively). There is a substantial difference in the carbonatitic melt composition within the trap and peridotite, with the latter having dolomitic Ca/(Ca + Mg) ratios of 0.38–0.60 (Fig. 4A). The conjugate silicic melts are high in SiO_2_ (53.3–84.5 wt%), Al_2_O_3_ (10.9–20.3 wt%), and have extreme K_2_O contents av. 9 wt% (1.1–12.7 wt%) (Fig. 4B,C). Most of the silicic melt compositions are characterized by lower totals than 100 wt% due to the high volatile contents. Analyses with totals below 80 wt% were rejected.

**Figure 5:**
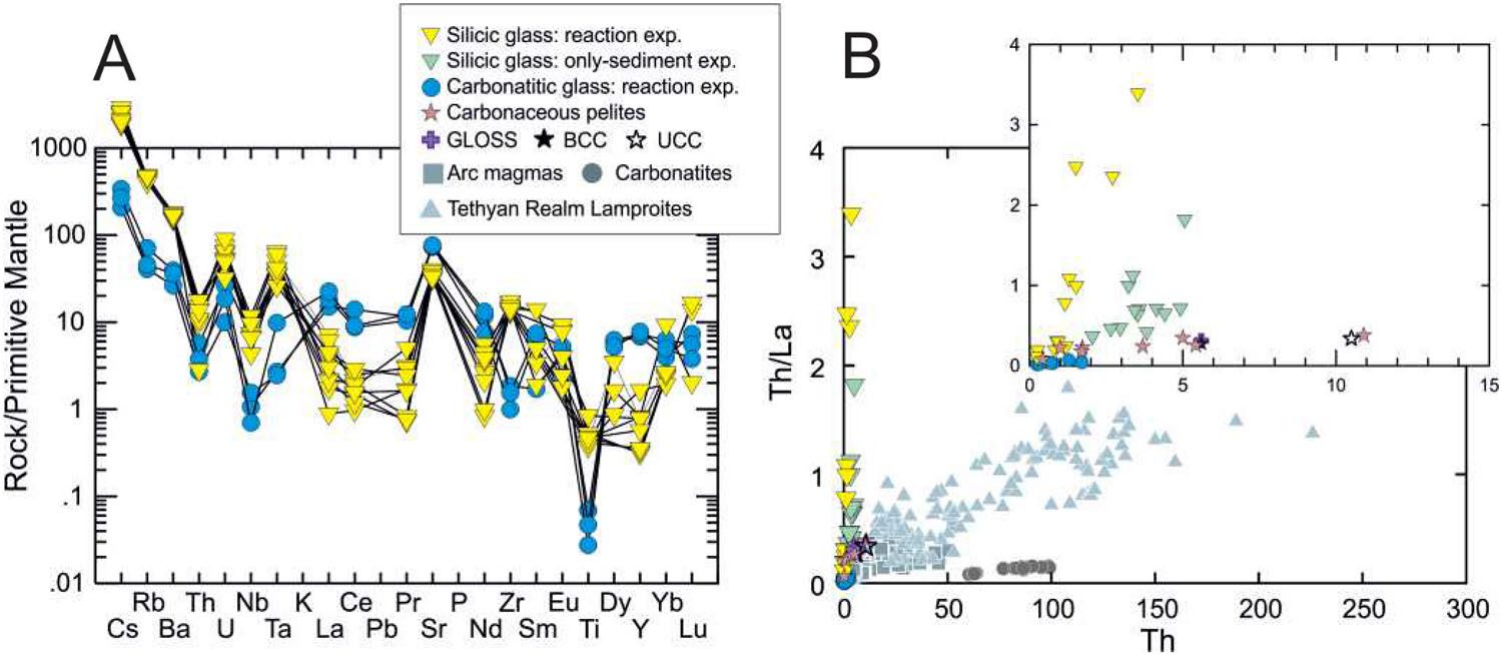
(**A**) Primitive mantle normalized trace element patterns of the representative carbonatitic and silicic glasses from reaction experiments between sediment and peridotite. Primitive mantle value is from^52^. (**B**) Th vs. Th/La diagram for the carbonatitic glasses from reaction experiments with silicic glasses from only-sediment and reaction experiments in this study. Carbonaceous pelite^6^, global subducting sediment (GLOSS^1^), bulk continental crust (BCC^53^), upper continental crust (UCC^53^), arc magmas (Pontide arc ultrapotassic rocks^22^), carbonatites (Mt. Vulture Carbonatites^54^), Tethyan Realm Lamproites^28^ are also plotted for comparison.

Two conjugate melts display trace element variations typical of carbonatitic and silicic melts; the silicic glass is enriched in LILE including Th and U, as well as Ti, Nb, Ta and Zr compared to the carbonatitic glass which is enriched in REE (with more intense La fractionation from the rest REE), Sr and P (Fig. 5A). These variations are particularly evident in specific trace element ratios such as Rb/Sr and Th/La which are more than tenfold higher in the silicic glasses compared to the carbonatitic glasses (Fig. 5B). This trace element behaviour aligns with partition coefficients established for conjugate carbonatitic and silicic melts in the water-bearing systems^46^, serving as strong evidence for the immiscible nature of the two melts.

### Melt-peridotite interaction and formation of metasomes

**Figure 6:**
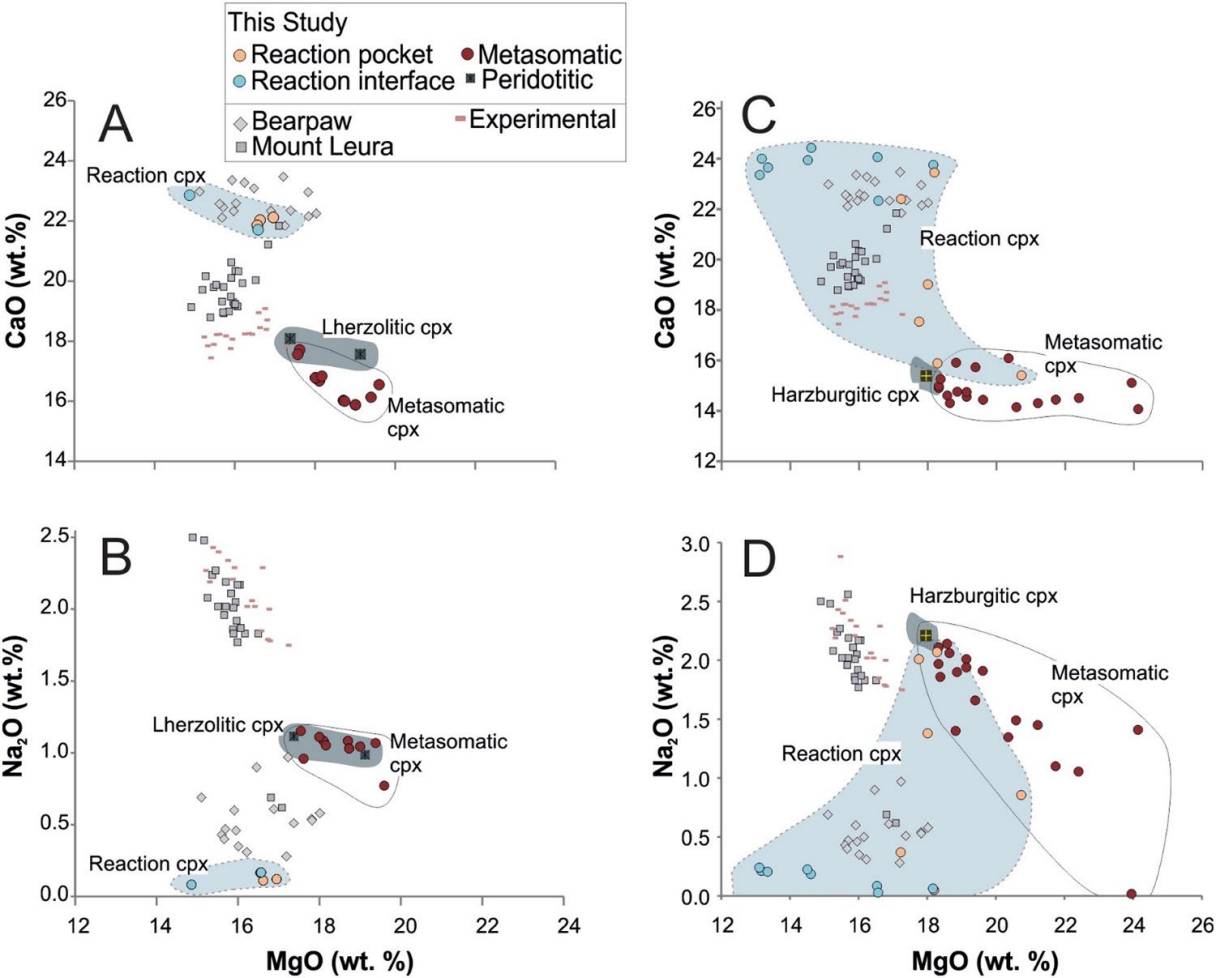
MgO (wt%) vs. Na_2_O and CaO (wt%) variation diagrams for the clinopyroxene subgroups in lherzolite-sediment (**A**, **B**) and harzburgite-sediment (**C**, **D**) reaction experiments. For comparison, clinopyroxene measurements from the synthetic lherzolite and harzburgite prepared for this study are plotted. The clinopyroxene data from other reaction experiments^48^, as well as natural xenoliths of Bearpaw^55^ and Mount Leura^56^ are plotted.

There is no systematic difference between the lherzolitic and harzburgitic portions of the experimental charges in terms of the metasomatic mineral association resulting from the melt-peridotite interaction in our experiments: wehrlitization of peridotite with the replacement of orthopyroxene (Opx) by clinopyroxene (Cpx) represents the universal process, which is most intense in the area closest to the sediment-peridotite interface (Fig. 2A,C). Opx and Cpx grains demonstrate significant disequilibrium textures, especially in the proximity of the sediment-peridotite interface, in contrast to spinel and olivine which are the least affected phases displaying no distinguishable textural changes (Fig. 2B). Cpx is the most abundant newly formed and metasomatic phase in the reaction experiments. According to its relation with Opx, it can be grouped as follows:

- Reaction Cpx (Cpx-r, pale grey, homogeneous, mantling Opx, up to 10 μm) comprises a rim around Opx grains, partially resorb and detach them from the peridotite portion (Fig. 2A, e.g. see near the left wall of the experimental charge). Moreover, this type of Cpx occurs in the clinopyroxene + phlogopite ± amphibole (Cpx-Phl-Amph) veins and pockets (Fig. 2B–D). In lherzolite-sediment experiments, it is generally diopsidic (Mg# = 0.88–0.89; Wo_45___49_En_45___48_ Fs_6_) with extreme enrichment in CaO up to 23.8 (wt%) and has low contents of MgO (14.5–16.9 wt%) and Na_2_O (0.1–0.2 wt%) (Fig. 6A,B). However, the Cr_2_O_3_ contents in Cpx from the reaction pockets are higher (0.46–0.51 wt%) than in the Cpx from the interface (0.16–0.21 wt%). On the contrary, in harzburgite—sediment reaction experiments, Cpx-r is augite (Mg# = 0.91–0.92; Wo_36–41_ En_54–58_ Fs_44–6_) with high Cr_2_O_3_ contents (2.0–2.7 wt%) whereas Cpx from the interface display variation between augite and diopside with lower Cr_2_O_3_ contents (0.04–1.5 wt%).
- Metasomatic Cpx (Cpx-m, dark colour, homogenous, euhedral crystals up to 20 μm) grains embay Opx grains due to the melt invasion, dissolution and recrystallization processes (Fig. 2B,C). The Cpx-m grains in lherzolite-sediment experiments are augitic and similar to Cpx from the unreacted peridotite portions (Mg# = 89–90; Wo_35–39_ En_55–58_ Fs_6–7_, CaO 15.8–17.7 wt%, Na_2_O 0.8–1.1 wt%, Cr_2_O_3_ 0.2–0.4 wt%; Figs. 2A,B and 6A,B). The same type of Cpx in harzburgite-sediment experiments is similar (Mg# = 0.91–0.93; Wo_27–36_En_58–66_ Fs_5–7_, CaO 14.2–16.1 wt%), but shows slightly elevated MgO contents up to 24.1 wt% (Fig. 6C). They also display variation in Cr_2_O_3_ (0.4–2.6 wt%) and Na_2_O (0.02–2.14 wt%) (Fig. 6D).

In addition to the newly formed Cpx (Cpx-r), phlogopite and amphibole are found as veins and domains together with dolomitic melt pockets within the peridotite (Figs. 2B and 3). Phlogopite forms small bladed and/or acicular crystals (largest 7 × 0.2 μm) (Fig. 2B). They are generally low in Ti (TiO_2_ < 0.8 wt%) and in Cr (Cr_2_O_3_: 0.03–0.5 wt%) and their Mg# is in the range between 85 and 92. The phlogopite crystals belonging to lherzolite-sediment reaction experiments (E10 and E15) display K_2_O contents up to 7.8 wt% and Cr_2_O_3_ between 0.4 and 0.5 wt% with Mg# ranging 90–93. In contrast, phlogopite from harzburgite-sediment reaction experiments (E10 and E17) has broader variation in Mg# (85–93) and more elevated contents of K_2_O (8.3–9.9 wt%) with lower contents of Cr_2_O_3_ (0.03–0.15 wt%) (Supplementary Data File I—Fig. 1). Amphibole in lherzolite-sediment reaction experiments is pargasitic with high Mg# = 91. The composition of the accompanied carbonate minerals was not identified but elementary maps reveal their high Ca and low Si contents (Fig. 3).

In summary, there is no systematic difference in the mineral assemblages forming metasomes within peridotite of different extents of depletion, and only their mineral composition reflects the more enriched (higher Ca, Al and Ti) character of the lherzolitic vs. harzburgitic host.

## Discussion

Our results provide a comprehensive examination of the intricate processes underlying the interaction between sediment-derived silicic-carbonatitic melt(s) and peridotite. Specifically, we gain valuable insights into how peridotites of varying fertility (lherzolite vs. harzburgite) interact with the melt through the crystallization of metasomatic minerals. In all our experiments, whether the peridotite is lherzolitic or harzburgitic in composition, we consistently observe the formation of vein-like structures of phlogopite-wehrlite and clinopyroxenite alongside the crystallization of dolomite and/or dolomitic glass. These features are interpreted as fundamental stages in the formation of metasomes^57^. The findings from the melt trap are particularly noteworthy. Our results indicate that under the conditions of our experimental setup, carbonate-rich siliciclastic sediments have the potential to generate conjugate carbonatitic and highly K-enriched silicate melts through a process of liquid immiscibility. This intriguing phenomenon involves their physical separation during the melting of carbonaceous pelites. This discovery offers a novel perspective on the role of mantle metasomatism and highlights the remarkably reactive nature of the recycling of carbonate-rich sediments. We utilize our observations regarding the geochemical characteristics of the three sections within our experimental capsules (melt trap, sediment, and peridotite) to discuss the extent to which our data contribute new constraints to the understanding of the petrogenesis of AHOB UP lavas, particularly the Si-undersaturated ones. After discussing the significance of the occurrence of conjugate carbonatitic and silicic melts, we address the issue of the kinship of Si-saturated and Si-undersaturated mantle-derived ultrapotassic melts in the light of the fractionation of several canonical trace element ratios such as Th/La and Sm/La. In a comprehensive view, the unique interplay of the processes of peridotite-melt interaction with the potential physical separation of the carbonatitic and extremely K-enriched silicate melts, including an integrated metasomatic response to these processes, will be crucial for the compositional evolution of these extremely alkaline melts and their mantle sources.

### New constraints on the petrogenesis of AHOB UP lavas

AHOB volcanic associations are generally characterized by universal enrichment of potassium coupled with invariably high incompatible trace element contents and isotopic compositions approaching values typical of the Earth’s continental crust^4–6,17–19^. In more detail, two compositionally different ultrapotassic volcanic series are recognized including the Si-saturated leucite-free series with lamproites and shoshonites as the most primitive lavas^58^, and Si-undersaturated leucite-bearing series with kamafugites as the most primitive lavas^58–61^. Experimental data suggest that the primary melts of Si-saturated series require high-degree melting of a phlogopite-bearing mantle source^3,48,57,62–67^. On the other hand, ultrapotassic Si-undersaturated primary melts will be sourced in the peridotite fluxed by the volatile components H_2_O and CO_2_, alternatively, in the wehrlitic mantle with the presence of metasomatic phases such as dolomite, phlogopite and amphibole^68–73^. A single experimental study investigated the interaction between limestone and peridotite at upper mantle conditions, ultimately producing alkaline reaction melts^32^.

Our experiments confirm previous studies, indicating that wehrlitisation results from the interaction between the melt of sediment with peridotite due to carbonatite metasomatism (e.g.,^12,14,74,75^). Moreover, the immiscible carbonatitic and silicic melts which released from the carbonaceous pelite interact with peridotites to form low-density assemblages of several hydrous minerals and dolomite within werhlite. The mineral assemblage produced, that is, a wehrlitic peridotite with phlogopite, amphibole and carbonate minerals/glass (Fig. 3) closely matches proposed mantle source compositions for orogenic Si-undersaturated ultrapotassic magmatism accounting for potassium enrichment and silica depletion^44,56,73,76^. The resemblance between the composition of observed metasomatic minerals and examples of mantle metasomatism from mantle xenoliths is striking. The phlogopite in our experiments exhibits low Ti and Cr contents, comparable to those in similar reaction experiments and natural xenolith samples^48,56^ (Supplementary Data File I—Fig. 7). Finally, the high Sr/Ba and low Th/U (well below the crustal average) observed in carbonatitic glass are typical characteristics of silica-undersaturated UP lavas in central Italy^59,77^.

In summary, our experiments demonstrate that phlogopite, pargasite and carbonate minerals can grow within the mantle wedge as a consequence of slab-fore arc interaction, resulting in metasomatic domains within the depleted peridotite mantle. Activation of these domains, which display internal heterogeneity on scales similar to those of melting and magma extraction (i.e. metres to kilometres), as suggested by isotopic data^5^ will form silica-undersaturated ultrapotassic melts if the degree of partial melting is not too low^78,79^.

### The Th/La conundrum revisited

Two distinct types of ultrapotassic volcanic series are traditionally believed to originate from separate mantle sources: ultrapotassic Si-saturated series are thought to stem from primary melts requiring a phlogopite-pyroxenitic mantle source^3,48,57,62–66^ whereas ultrapotassic Si-undersaturated primary melts will be sourced in the wehrlitic mantle with the presence of metasomatic phases such as dolomite, phlogopite and amphibole^68–73^. They were thought to share only a general connection to continental crustal sedimentary material responsible for the metasomatic transformation of their mantle source. This rigid dichotomy has been exemplified by the bimodal character of the Italian orogenic magmatism where these two lava types are spatially and timely separated (Roman vs. Tuscan Magmatic Province; e.g.^77^ and references therein). However, several recent studies have revealed significant kinship of the two distinct types of ultrapotassic series, supported by the coexistence of both melt types and their derivative minerals in Latera and San Venanzo volcanoes^4,80^. This has been interpreted as being a result of a shift from pelitic to carbonate-rich sediment flux recycled within the mantle below the Apennines sediments (carbonate-rich vs. pelitic), being able to transform the mineralogy of the sub-arc peridotite on laterally small scales^4^. The interaction of the sediments with the peridotite resulted in mantle portions of contrasting compositions, implying that multiple instances of recycled sedimentary material are operating in this region of the Italian arc. This notion aligns with a recent proposal that recycled carbonate sediments contribute to the unique isotopic signatures observed in Mediterranean lamproites (Si-saturated series), termed "Mg–Zn isotopic decoupling"^81^.

In addition to trace element enrichments, Si-saturated AHOB lavas (lamproites) display an intriguing positive correlation between the Th/La and Sm/La ratios, which is not demonstrated by the Si-undersaturated leucite-bearing series. This enrichment pattern is inconsistent with the typical mantle source metasomatized by slab-derived components, as such a correlation is not observed in arc magmas, and the Th/La ratio is generally not greater than 0.58. Moreover, this pattern is not seen in the crust, mantle, or most mantle-derived melts, suggesting an unconventional source or process at play. In a comprehensive examination of this issue^28^, it was proposed that the intriguing Th/La and Sm/La increase is mineral-controlled, being evidence for the existence of an ancient component (referred to as SALATHO) enriched in lawsonite. This component, stored within the recycled mélange, could potentially account for the high Th/La and Sm/La ratios. However, the extent to which other components, such as high K and distinctive isotopic signatures, contribute to the enrichment in AHOB ultrapotassic lavas remains only a partially addressed aspect.

Our experiments provide arguments to build an alternative explanation for this paradox. It is the possibility of immiscibility between carbonatitic and silicic melts at PT conditions close to those in the fore-arc regime and their potential physical separation, which opens a new perspective on trace element fractionation not taken into account previously. Conjugate carbonatitic and silicic melts generated by melting carbonaceous pelites exhibit substantial differences in trace element concentrations and ratios. Notably, the Th/La ratio in the hydrous and K-rich silicic glass is up to five times higher than in the carbonatitic glass due to the intense fractionation of these elements. This fractionation aligns with experimentally determined partition coefficients between carbonatite and silicate melts in hydrous K-rich silicic systems^46^. While this aligns with the signature recognized in Si-saturated AHOB lavas, the fractionation of Sm/La ratio is less intense (Supplementary Data File II). Nevertheless, we propose that this geochemical signature can be transferred to the silicate portion of the mantle, particularly within phlogopite-clinopyroxene-rich metasomes, which could then exhibit this unusual geochemical signal. This transfer may be facilitated by the separation of silicate melt from carbonatitic melt, carrying the necessary ingredients to create a metasomatized source for lamproites: high potassium content, extremely high Th/La ratios (variable but high Sm/La ratio) and distinct isotopic signatures.

In the scenario of subduction-induced sediment recycling and cyclic metasomatism at the slab-(fore-arc) mantle interface, alkaline fluids migrate upward through melting, solidification/freezing, and reaction cycles as subduction progresses. We speculate that the melting of carbonaceous pelite yields alkaline dolomitic and hydrous silicic-potassic melts. Infiltration of these melts into depleted mantle might lead to decoupled metasomatic events. The initial metasomatism by the dolomitic melt results in a trend from harzburgite to olivine-rich wehrlite. Further melting of the metasomes in a region containing dolomite-bearing phlogopite wehrlites, produces kamafugites without a Th/La anomaly. Interaction of the silicate melt with peridotitic mantle leads to silica and potassium enrichment, and depletion in HFSEs relative to LILE, within phlogopite clinopyroxenites. If the two melts are segregated within the mantle based on viscosity and permeability differences, infiltration of the silicate melt enriches metasomatized rocks in clinopyroxene and phlogopite, ultimately giving rise to lamproites exhibiting the Th/La anomaly.

## Conclusion

We can draw the following conclusions from our study:

- In a series of 2 GPa experiments in a piston-cylinder apparatus at 800 and 850 °C, we combine carbonaceous pelites with either harzburgite or lherzolite in the presence of water (20 wt% of the sediment), simulating the crust-mantle interactions and formation of mantle metasomes in fore-arc mantle conditions.
- Two conjugate melts, that is, carbonatitic and ultra-high-K silicate melts are produced, representing strong metasomatizing agents.
- The produced metasomes consist of clinopyroxene + phlogopite ± amphibole ± carbonate minerals that would be able to produce Si-undersaturated ultrapotassic melts (leucitites and kamafugites) during the further stages of orogenesis resulting in the post-collisional reactivation of the accreted fore-arc mantle.
- Two conjugate melts demonstrate a strong potential for incompatible trace-element fractionation, with silicate portions driving high Th/La and LILE/HFSE, as observed in Si-saturated utrapotassic lavas.

### Supplementary Information

Supplementary Information 1.

Supplementary Information 2.

## Data Availability

All data generated or analyzed during this study are included in this published article and its supplementary information files.
