# Supplementary material for: Experimental production of K-rich metasomes through sediment recycling at the slab-mantle interface in the fore-arc
Source: Sci Rep. 2023 Nov 10;13:19608. doi: 10.1038/s41598-023-46367-7 (PMC10638307; doi:10.1038/s41598-023-46367-7)
Supplement: Supplementary file 1 — Supplementary Information 1. [file 41598_2023_46367_MOESM1_ESM.docx]

Supplementary Data File I

**Experimental production of K-rich metasomes through sediment recycling at the slab-mantle**

**interface in the fore-arc**

Fatma Gülmez, Dejan Prelevi´ć, Michael W. Förster, Stephan Buhre, Jennifer Günther

# Methods

## Experimental Procedure

The experiments in this study have been conducted using the rapid quench piston cylinder apparatus, in the Experimental Petrology Laboratory at Johannes Gutenberg University Mainz (JGU). Further information and technical details on the identical apparatus manufactured by GUKO Sondermaschinen–bau GmbH, Uslar, Germany, and installed in the School of Natural Sciences at Macquarie University, Sydney, Australia can be found in the recent study (Ezad et al., 2023). The first two runs were performed with modular capsules at 850 °C and 800 °C/2 GPa and the following runs with simple design repeated under the same conditions separately for the combinations of the harzburgite + sediment and the lherzolite + sediment. Given the challenge to produce and specially to measure the glass compositions, beside reaction experiments, we performed the “only-sediment experiments” at two different temperatures, in addition to six reaction experiments, in which there is no peridotite. Only-sediment experiments (6 days) have been conducted under the same conditions as reaction experiments (15 days) but for a shorter duration. Available measurement data of the acceptable quality have shown that there are no systematic differences in the glass compositions between the reaction- and only-sediment experiments.

## Analytical Methods

The polished experimental charges were imaged and the mineral and melt phases were analyzed using the JEOL JXA 8200 Superprobe electron probe microanalyzer at (EPMA) JGU. Operating conditions were set to 20 kV accelerating voltage, 20 nA beam current and 2 μm beam diameter. Synthetic and natural minerals were used as reference materials. To better document reactions between peridotite and sediment under given conditions backscattered electron images of the reaction zones and metasomatic portions were produced. We identified the melt composition by measuring glasses that are devoid of quench crystals throughout the experimental charges. Where sufficiently large melt pockets are absent, element distribution maps were produced to document the glass compositions (Figure 1). The diamond trap drained the initial melts from the sediment and helped us with the accurate determination of unfractionated melt compositions.

Trace element analyses of the glasses were performed by LA-ICP-MS at the University of Mainz using an ArF EXCIMER-laser (193 nm wavelength, NWR193 system by esi/NewWave) coupled to an Agilent 7500ce ICP-MS system according to the standard procedure described in described in detail in one of our previous studies (Gülmez et al. 2016). The measurements were calibrated based on the NIST 610, NIST 612 and BCR glass reference materials. Iron-loss from the system to the Au-Pd outer capsule was calculated and found to be insignificant based on mass balance (Supplementary Data File II). We also employed mass balance calculations to justify the visually estimated modal proportions of the mineral and melt phases.

# Starting material composition

Starting materials were composed of the synthetic mantle and natural lithologies. To prepare the peridotite end-members, high-purity, commercially available oxide powders (SiO_2_, TiO_2_, Al_2_O_3_, Fe_2_O_3_, K_2_O, Na_2_O, MgO, CaO, Cr_2_O_3_, NiO and MnO) were mixed with ethanol in an agate mortar and pestle for more than 2h and were synthesized at 1250 ^o^C/2 GPa according to the standard procedure by Rapp et al. (1999). Fertile peridotite is a natural spinel lherzolite sample (KLB-1) from Kilbourne Hole, USA (Rapp et al., 1999), while the depleted peridotite (AVX) is a harzburgite from Volvoyam Volcanic Field, Kamchatka Arc (Kepezhinskas et al., 1995). We have also calculated their bulk compositions using the electron probe micro analysis of mantle phases (olivine, orthopyroxene, clinopyroxene, spinel) and their modal proportions in the synthetic harzburgite and lherzolite. A comparison between the re-calculated and measured compositions for the KLB-1 (Table [1)](#_bookmark0) and AVX (Table [2)](#_bookmark1) suggests that the synthetic mantle materials are analogs of the natural lherzolite (KLB-1) and harzburgite (AVX).

The natural sediment sample is a carbonaceous pelite - marlstone from the Apennine Region (SD48), which is geochemically characterized by Avanzinelli et al (2008) and Conticelli et al. (2009) and discussed as one of the potential end members involved in crustal recycling within the mantle source of Italian ultrapotassic magmatism.

We have reconstructed the bulk composition of the carbonaceous pelite by performing mass-balance calculations based on the composition of the residual minerals and extracted melts which were obtained from the reaction and the only- sediment experiments (Table [3).](#_bookmark3) We excluded the trapped melts and employed the residual phases and glasses for the calculations, considering that the initial melts are volumetrically negligible and compositionally inconsistent with the glass in the crustal part of the experimental charges.

**Table 1:** The average composition and modal proportions of the mantle minerals. Comparison of the calculated lher- zolite composition with the bulk analysis of the natural lherzolite sample. Ol: olivine, sp: spinel, opx: orthopyroxene, cpx: clinopyroxene

| Minerals | Ol | Sp | Opx | Cpx | Calculated  Lherzolite | Natural  Lherzolite | Deviation |
| --- | --- | --- | --- | --- | --- | --- | --- |
| SiO_2_ | 40.37 | 0.19 | 53.09 | 52.39 | 44.55 | 44.59 | 0.09 |
| TiO_2_ | 0.02 | 0.12 | 0.16 | 0.28 | 0.09 | 0.16 | 42.05 |
| Al_2_O_3_ | 0.11 | 64.11 | 6.86 | 5.88 | 3.94 | 3.59 | 9.81 |
| Cr_2_O_3_ | 0.06 | 3.33 | 0.28 | 0.30 |  |  |  |
| FeO | 9.78 | 8.54 | 6.27 | 3.89 | 7.99 | 8.1 | 1.32 |
| NiO | 0.24 | 0.31 | 0.10 | 0.10 |  |  |  |
| MnO | 0.11 | 0.08 | 0.12 | 0.15 | 0.12 | 0.12 | 2.15 |
| MgO | 49.38 | 22.86 | 31.66 | 18.26 | 39.75 | 39.22 | 1.36 |
| CaO | 0.15 | 0.03 | 1.41 | 17.82 | 3.11 | 3.44 | 9.57 |
| Na_2_O | 0.01 | 0.02 | 0.14 | 1.05 | 0.20 | 0.3 | 32.83 |
| K_2_O | 0.00 | 0.00 | 0.00 | 0.00 | 0.00 | 0.02 | 100.00 |
| Total | 100.23 | 99.59 | 100.07 | 100.10 | 99.76 | 99.54 | 0.22 |
| Modal |  |  |  |  | R | 0.9999 |  |

Proportions 0.58 0.02 0.25 0.15 R^2^ 0.9998

The calculations were also performed to double check the visually estimated proportions of the residual phases. The reaction is as follows:

Sediment + water = calcite_0.7_ + quartz_0.09_ + feldspar_0.04_ + garnet_0.04_ + mica_0.03_ + amphibole_0.02_ + epidote_0.01_ + silicate melt_0.05_ + carbonate-silicate melt_0.02_

Except the TiO_2_ and Al_2_O_3_ deviations, each element oxide in the reconstructed composition is compatible with the measured sediment composition. The calculations confirm that the ratio of the melt extraction from the sediment is of 7 wt.%.

**Table 2:** The average composition and modal proportions of the mantle minerals. Comparison of the calculated harzburgite composition with the bulk analysis of the natural harzburgite (see the Table [1](#_bookmark0) caption for abbreviations).

| Minerals | Ol | Sp | Opx | Cpx | Calculated  Harzburgite | Natural  Harzburgite | Deviation |
| --- | --- | --- | --- | --- | --- | --- | --- |
| SiO_2_ | 40.62 | 3.21 | 57.33 | 53.24 | 44.43 | 44.48 | 0.12 |
| TiO_2_ | 0.01 | 0.03 | 0.00 | 0.01 | 0.01 |  |  |
| Al_2_O_3_ | 0.02 | 17.27 | 0.70 | 3.69 | 0.65 | 0.69 | 6.38 |
| Cr_2_O_3_ | 0.19 | 48.61 | 0.29 | 2.64 |  |  |  |
| FeO | 8.87 | 13.54 | 5.14 | 3.56 | 7.87 | 7.43 | 5.90 |
| NiO | 0.45 | 0.21 | 0.16 | 0.12 |  |  |  |
| MnO | 0.11 | 0.14 | 0.10 | 0.08 | 0.11 | 0.12 | 8.68 |
| MgO | 49.99 | 18.33 | 34.27 | 17.96 | 44.47 | 43.85 | 1.40 |
| CaO | 0.13 | 0.01 | 1.32 | 15.39 | 0.88 | 0.91 | 2.77 |
| Na_2_O | 0.03 | 0.02 | 0.20 | 2.21 | 0.14 | 0.31 | 55.71 |
| K_2_O | 0.01 | 0.01 | 0.01 | 0.00 | 0.01 | 0.04 | 85.86 |
| Total | 100.43 | 101.37 | 99.51 | 98.90 |  |  | 0.74 |

R 0.9999

| Modal |  | | | |
| --- | --- | --- | --- | --- |
| Proportions | 0.7 | 0.02 | 0.25 | 0.03 |

R^2^ 0.9999


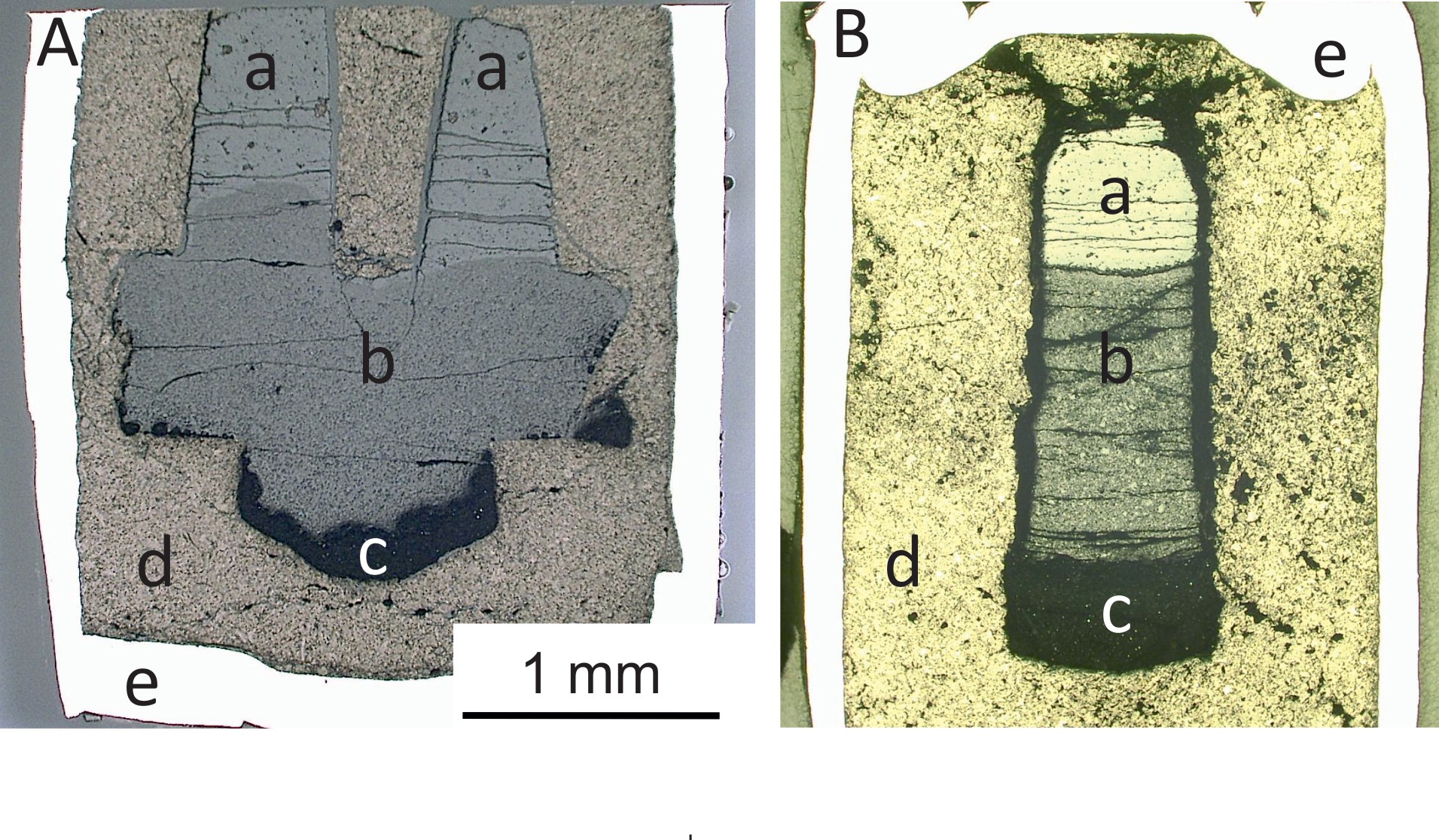


**Figure 1:** Photos of epoxy-embedded and polished mounts showing the different designs under reflected light. a) Modular capsule design, b) Simple capsule design (a: mantle, b: crust, c: diamond trap, d: graphitic inner capsule, e: Au-Pd outer capsule).

# Capsule Design

The reaction experiments were performed in two different types of capsule designs (Fig. [1):](#_bookmark2)

1. The simple capsule design combines either synthetic lherzolite or harzburgite with the carbonaceous pelite, in which these rock types make up two halves of each inner graphite capsule with a peridotite/sediment ratio of 2/5 placed within an outer Au-Pd capsule (Fig. [2b).](#_bookmark4) The bottom part of the inner capsule was loaded with a layer of a fine-grained 10*µ*m diamond trap (Fig. [2).](#_bookmark4) The lherzolite + sediment and the harzburgite + sediment reaction experiments were performed separately and brucite (Mg[OH]_2_) is used as the water source.
2. The modular design includes an inner graphite capsule with a lower large hole (3.0 x 2.5 mm) which hosts the carbonaceous pelite fluxed with H_2_O (% 20 wt. of sediment), and an upper half with two holes, each (0.9 x 1.5 mm) that are loaded either with harzburgite or lherzolite (Fig. [3).](#_bookmark5) The mantle/sediment ratio is 3/5. This capsule design allows observing whether the fertile and depleted mantle lithologies respond to sediment melts similarly, under the same conditions.

In both design types, the inner graphitic capsules are loaded within outer Au-Pd capsules of 3.6 x 7.0 mm which are carefully sealed to minimize water loss. The diamond trap is used to capture initial melts/fluids from the melting of sediment in both capsule designs (Fig. 1). This is made by the geometry of the experiments, in which the peridotite is placed on the top of the capsule, whereas the melt-trap on the bottom. That means that the melts collected in the diamond trap should most closely represent the partial melts of the sediment, and not the melt reacted with the peridotite. Given the challenge of producing and especially measuring the glass composition, besides reaction experiments, we performed also the “only-sediment experiments” at two different temperatures, in which there is no peridotite. Available measurement data of acceptable quality have shown that there are no systematic differences in the glass compositions between the reaction- and only-sediment experiments.


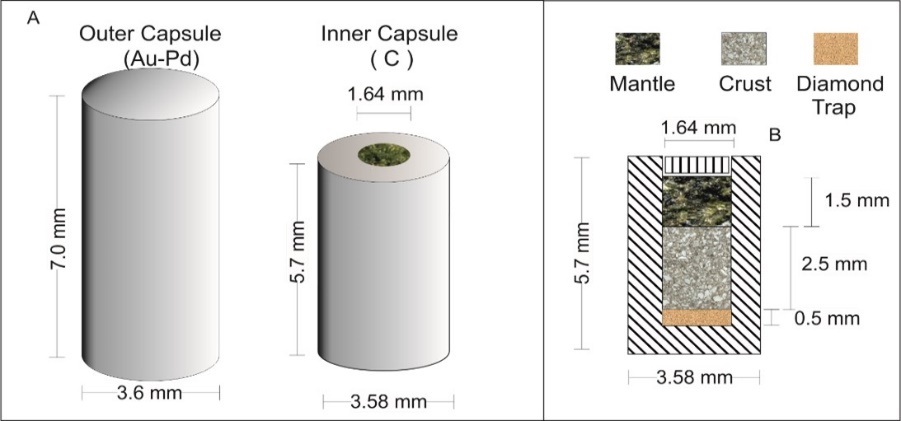


Figure 2: Dimensions for simple capsule designs (UGC: Upper Graphitic Capsule, LGC: Lower Graphitic Capsule). Mantle/crust ratio is of 3/5.


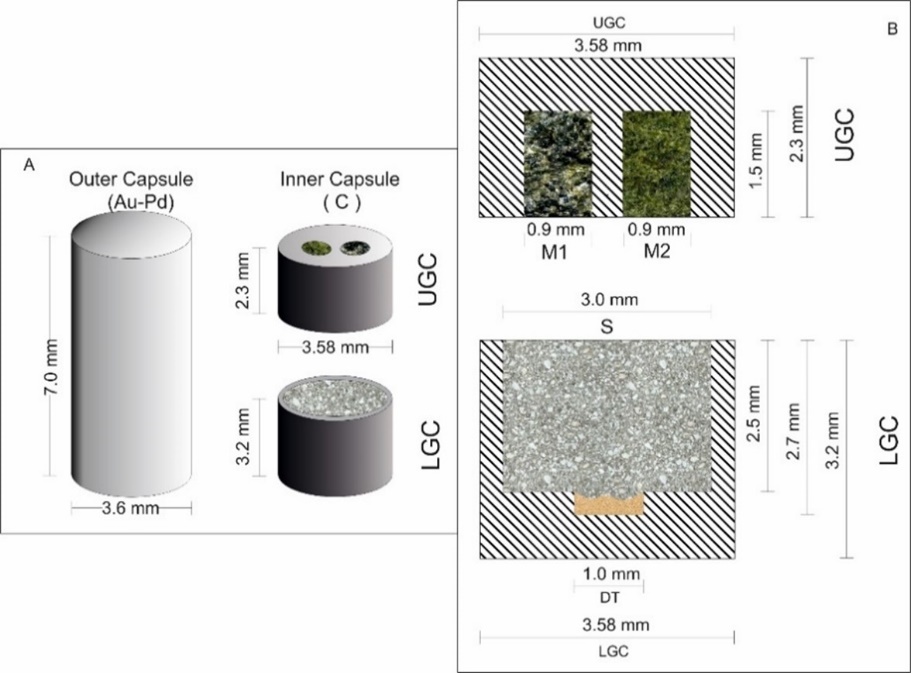


Figure 3: Dimensions for modular capsule design (UGC: Upper Graphitic Capsule, LGC: Lower Graphitic Capsule). Mantle/crust ratio is of 1/3.

3

Table 3: Mass balance calculations to confirm the proportions of the residual phases and glass. We obtained several types of glass compositions resembling silicic and carbonatitic melts and mixture of melts as carbonated silicate melt. No carbonatitic melt is observed within the sediment portion.

| Sediment | | Sediment | Sediment | Deviation |  | |  | Residual Phases | | | | | |  |
| --- | --- | --- | --- | --- | --- | --- | --- | --- | --- | --- | --- | --- | --- | --- |
| Composition | | Composition | Composition |  | Qz Cal | | Amp | Ep Mca Fsp Grt Sil Melt Mix Melt | | | | | |  |
|  | | Normalized to 100 | Reconstructed |  |  | |  |  | | | | | |  |
| SiO_2_ | 18.80 | 27.21 | 29.53 | 8.51 | 98.4 | 1.6 | 60.2 | 45.6 | 46.3 | 65.1 | 38.5 | 66.2 | 25.8 |  |
| TiO_2_ | 0.28 | 0.41 | 0.83 | 106.02 | 0.0 | 0.0 | 0.3 | 0.2 | 1.1 | 0.0 | 0.5 | 0.1 | 26.1 |  |
| Al_2_O_3_ | 6.70 | 9.70 | 6.23 | 35.80 | 0.0 | 1.2 | 4.6 | 28.0 | 27.3 | 19.0 | 20.5 | 14.3 | 6.5 |  |
| FeO | 2.52 | 3.65 | 3.22 | 11.66 | 0.0 | 2.1 | 6.8 | 2.2 | 2.2 | 0.1 | 14.2 | 0.1 | 0.4 |  |
| MnO | 0.11 | 0.16 | 0.17 | 9.50 | 0.0 | 0.1 | 0.1 | 0.0 | 0.0 | 0.0 | 0.8 | 0.0 | 0.0 |  |
| MgO | 1.24 | 1.79 | 1.63 | 9.00 | 0.0 | 1.2 | 8.7 | 0.4 | 1.8 | 0.0 | 2.6 | 0.0 | 0.1 |  |
| CaO | 37.84 | 54.77 | 55.56 | 1.44 | 1.2 | 53.1 | 20.0 | 22.6 | 7.0 | 1.5 | 21.2 | 0.4 | 26.5 |  |
| Na_2_O | 0.28 | 0.41 | 0.39 | 3.11 0.0 0.1 0.4 | | | | 0.0 | 0.9 | 4.1 | 0.1 | 0.9 | 0.0 |  |
| K_2_O | 1.28 | 1.85 | 1.82 | 1.62 0.0 0.1 0.2 | | | | 0.0 | 7.4 | 10.0 | 0.2 | 11.9 | 0.1 |  |
| Total | 69.09 | 100 | 100 | 0 99.8 59.9 101.6 | | | | 99.7 | 95.1 | 100.0 | 98.9 | 94.3 | 88.6 |  |
|  |  |  | R^2^ = 0.9975 | Proportions % = 9 70 2 | | | | 1 | 3 | 4 | 4 | 5 | 2 | *Total*  100 |

# Additional information for the melt and mineral compositions

##

## Melt Composition

###

### Carbonate melts

The only-sediment experiments (direct melting of carbonaceous pelite) produced carbonatitic melts with higher con- tents of Na_2_O (*∼* 2 wt.% water-free) than the sediment-peridotite reaction experiments, but they are represented by the lowest K_2_O / Na_2_O ratios and similar contents of CaO/Al_2_O_3_ with the infiltrated carbonate melts (Fig. 3b-c in the manuscript). The low alkali content of the trapped (av. Na_2_O: 0.04 wt.% and av. K_2_O: 0.18 wt.%) and infiltrated melts (av. Na_2_O: 0.04 wt.% and av. K_2_O: 0.03 wt.%) is characteristic of our reaction experiments due to the longer duration than only-sediment experiments (6 days). The K_2_O/Na_2_O ratios for the carbonatitic melts from the reaction experiments could be as high as 10, but obviously this is artificial, considering that their potassium content is as low as negligible (Fig. 3c in the manuscript).

### Silicate melts

Glass composition in natural mantle xenoliths and in experimental studies that conducted at various conditions representing shallow to deep mantle were well-documented to be in a great range between silica undersaturated and oversaturated (Andersen and Neumann, 2001 and references therein). The silicic melts in our reaction experiments display good correlation with the melt compositions from the previous reaction experiments conducted at 800-900^o^C [(Förster](#_bookmark14) et al., 2019). Nevertheless, the only-sediment experiments produced silicic melts with a lower SiO_2_ content (53.3 – 66.3 wt.%) which are similar to the melts from high temperature reaction experiments (1000-1100^o^C) and to melts from melting experiments conducted at a range of pressure and temperature conditions (2-5 GPa and 900-1300^o^C; Thomsen et al. 2008, Förster et al. 2019, Chunfei et al. 2021; These melts also tend to have relatively higher K_2_O (3.4 -12.7 wt.% av. 10.7 wt.%), Na_2_O (1.8 – 7.0 wt.% av. 3.9 wt.%) and lower ratios of K_2_O/ Na_2_O = 4 (Fig. 3c in the manuscript). This should also have resulted from the difference in duration between the sediment-peridotite reaction and only-sediment experiments, hence the measurements on the silicic melts are all from the diamond trap.

## Melt and Mineral Compositions

### Spinel

Spinel is the only phase which was not affected by the reactions. Despite this, the average spinel compositions in harzburgite which was calculated based on the spinel analysis on experimental runs and synthetic spinel, reveal large standard deviations from the initial compositions (R^2^ is as low as 0.91). This might be resulted from the mix analysis on the small grains. As a result of this, the compositions of the harzburgitic spinel shifted from the initial value of [(Mg_0.7_ Fe_0.3_) (Al_0.7_ Cr_1.2_ Fe_0.1_) O_4_] by decreasing in Cr# (35.4 – 79.0) with no significant change in Mg# (67.1 – 73.6). On the other hand, the composition of the spinels in the lherzolite portion of the experimental charges [(Mg_0.85_ Fe_0.15_) (Al_1.85_ Cr_0.15_)O_4_] has Cr# [Cr/(Cr+Al) × 100]: 3.8 – 5.3 and Mg# [Mg/(Mg+Fe) × 100]: 81.4 – 82.0, is principally the same as in the unreacted peridotite (Fig. [4).](#_bookmark6)

### Olivine

The interactions between low-silica melts and orthopyroxene can produce either olivine or clinopyroxene without visible evidence of metasomatism (Matveev et al. 2001), however we do not observe second-generation olivine formation or profound textural/compositional changes in the harzburgitic and lherzolitic olivines in our experiments. Olivine is the least affected phase in the reaction experiments. The grains are observed to be homogeneous and show no textural evidence of metasomatism, except just a few grains at the sediment-mantle interface. These grains are characterised by the devel- opment of sieve and spongy textures along their rims where reaction orthopyroxene growth has occurred due to the melt invasion and incongruent dissolution processes. Serpentinization may have affected the olivines compositionally without resulting in textural changes that could be identified with back scattered electron images. The forsterite content [Fo = Mg/(Mg+Fe) × 100] of the olivines belonging to lherzolite-sediment reaction experiments range from

89.5 to 91.1. Compared to the unreacted peridotite these grains are slightly enriched in CaO (0.18 – 0.25 wt.%). The olivines in harzburgite- sediment experiments show enrichment in Al_2_O_3_ (0.05 - 0.5 wt.%) but have similar CaO (0.03 – 0.31 wt.%) and forsterite contents (90.3 to 91.6) to the unreacted peridotite.


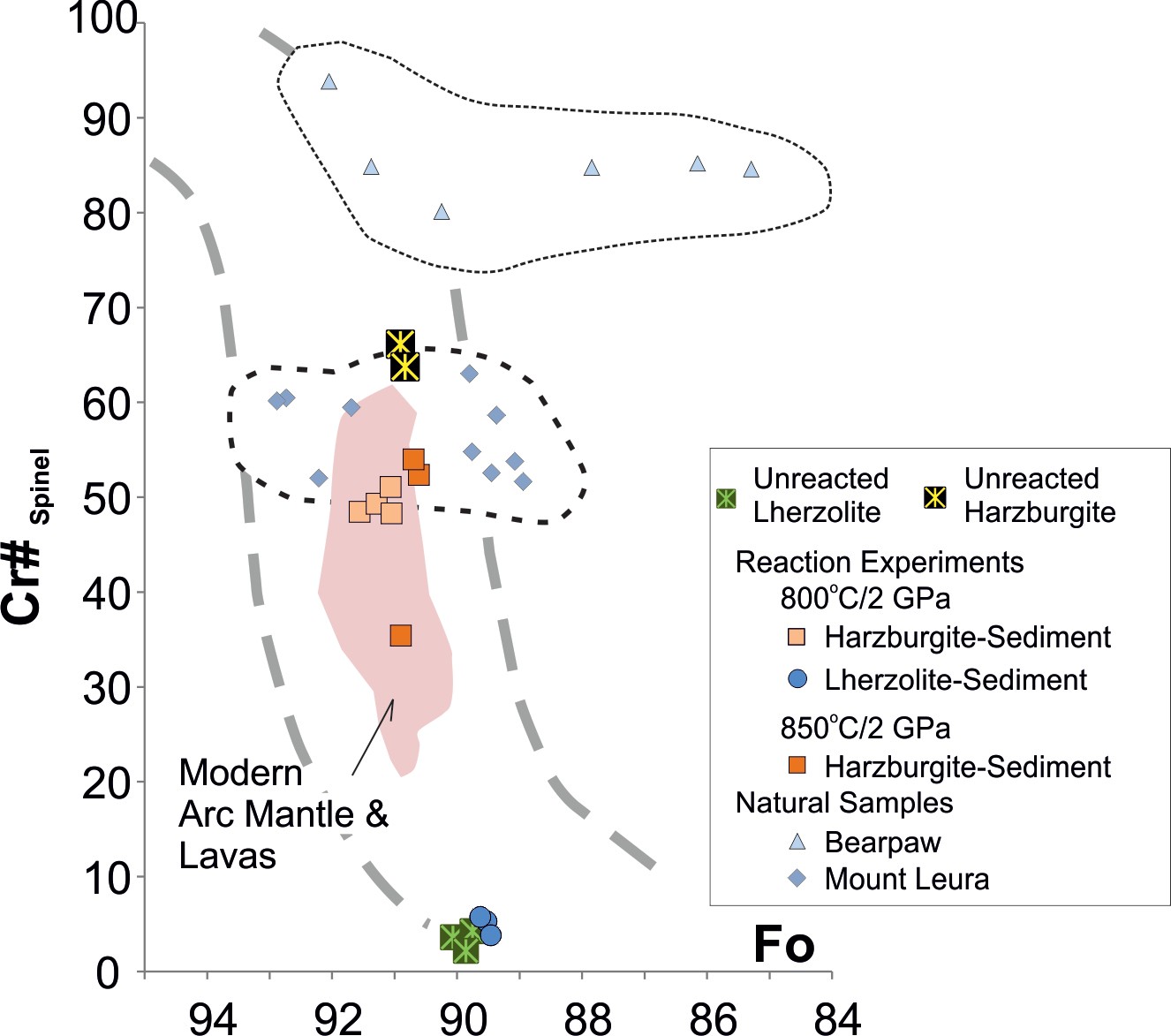


Figure 4: Average Forsterite content of olivine versus average Cr# of spinel from peridotite-sediment reactions ex- periments (averages calculated for each possible run). Dashed grey lines representing the olivine-spinel mantle array is from Arai (1994, 1992), modern arc mantle and lavas are from Prelević et al. (2013), Mount Leura and Bearpaw metasomatic peridotite samples are from Bonadiman et al. (2021) and Downes et al. (2004) respectively.

### Orthopyroxene

The mass balance calculations for metasome formation as well as the textural relationships of the phases in our reaction experiments confirm that the orthopyroxene is the major reactant phase leading the crystallization of the second-generation phases independent from the primary mantle composition. The metasomatic Opx grains in the lherzolite-sediment experiments show depletion in Al_2_O_3_ (down to 1.4 wt.%) with increases in Mg# (up to 93.5) and CaO (1.4 - 2.4 wt.%) (Mg# = 90 – 94; Wo3-5 En86-91 Fs6-10) whereas those in the harzburgite-sediment experiments (Mg# = 91 – 94; Wo_2-4_ En_89-91_ Fs_6-8_) tend to have elevated contents of Al_2_O_3_ (0.6- 3.8 wt.%) and similar contents of CaO (1.3-2.3 wt.%), when compared with the Opx grains from the unreacted peridotite (Fig. [6).](#_bookmark7)

### Clinopyroxene

The diopsidic clinopyroxene which is low in NaO due to the relatively low crystallization temperature at 800 - 850°C (wt.%) is the most abundant metasomatic mineral phase formed during our reaction experiments. This finding is in good agreement with the growing consensus that clinopyroxene-rich peridotites are of metasomatic origin as previously stated by Green and Wallace (1988) and carbonatitic melts infiltrating lherzolite at pressures *<*21 kbar at 950-1050^o^C result in replacement of lherzolite or harzburgite towards wehrlite compositions by removal of orthopyroxene and precipitation of pargasite in the presence of low H_2_O contents.

### Phlogopite

Phlogopite is an exotic mineral within the mantle that is thought to result from the interaction between refractory peridotite and either high-K silicic melts/ fluids (Wyllie and Sekine, 1982; Sekine and Wyllie, 1983) or asthenosphere with lithosphere (Menzies et al., 1987). In contrast, the previous studies focusing on the mantle phlogopite suggested that its crystallization occurs at high pressure (*>* 3 GPa) and temperature (*>* 1200^o^C; Poli, 2015; Mallik, 2016), our experiments together with the recent experiments (Förster et al., 2021) confirms phlogopite pyroxenite can grow at mantle wedges. The composition of the newly formed phlogopite crystals is best seen on Al_2_O_3_ vs Ti_2_O

and FeO with K_2_O vs. Mg# plots (Fig. 1a-c). The K/Na ratio in phlogopite and pargasite is an indicator of the origin of the metasomatising agent (Arai, 1986), therefore we have plotted K/Na ratios against Cr_2_O_3_ wt.%. Phlogopite shows substantial variation in K/Na from 5.5 to 160.5 in harzburgite-sediment reaction experiments whereas much more consistent (8.0 – 16.4) lherzolite-sediment experiments, without suggesting any further discrimination.


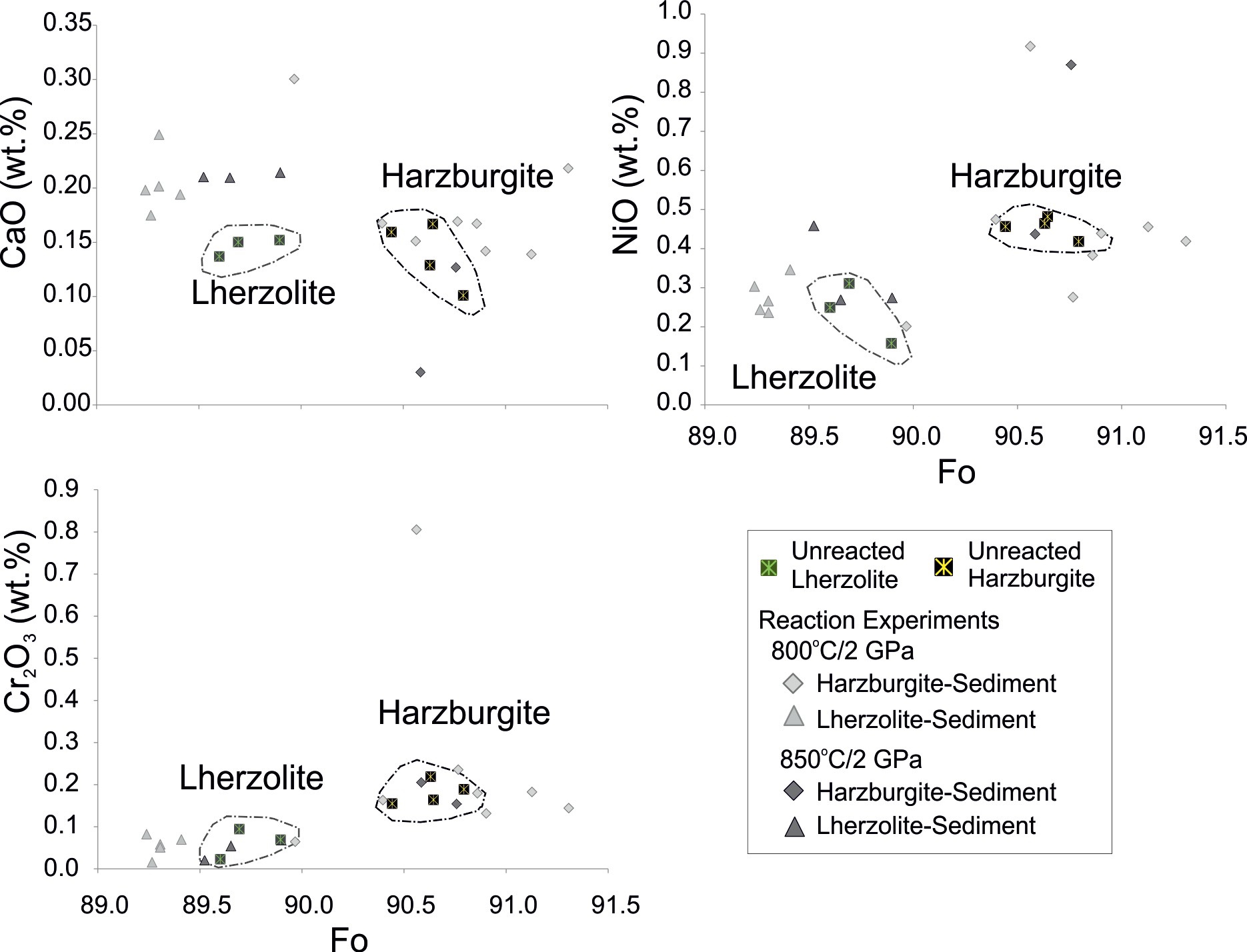


Figure 5: Forsterite vs. CaO (wt.%), NiO (wt.%), Cr_2_O_3_ (wt.%) diagrams showing slight shifts from original olivine compositions.


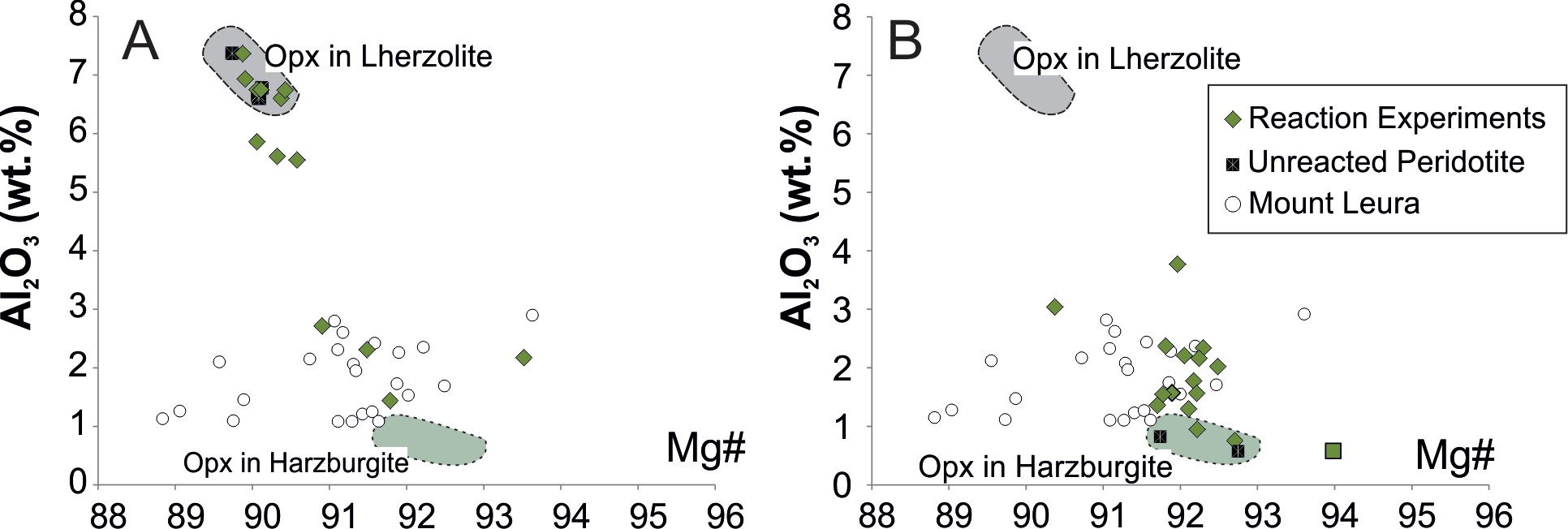


Figure 6: Mg# [Mg/(Mg+Fe) × 100] versus Al_2_O_3_ wt.% plot for orthopyroxene minerals from a) Lherzolite-sediment reaction experiments, b) harzburgite-sediment reaction experiments. Mount Leura represents the orthopyroxenes from phlogopite and amphibole bearing harzburgite xenoliths [^9^](#_bookmark17).

#

# Metamorphism of the carbonaceous pelite

Carbonaceous pelite (marlstone), undergoes progressive fluid-present metamorphism due to increasing temperature and pressure in the piston-cylinder. Residual phases consist mainly of calcite, quartz (coesite), epidote and amphibole. Apatite, rutile, sphene and pyrite crystals are accessory minerals in equilibrium with carbonate and silicate hydrous melts in our experiments. Garnet porphyroblasts are found restricted along a thin layer over the melt trap. Presumably, the breakdown of the hydrous phases of the pelite during the prograde metamorphism contributed to the total budget of water in the system. It is important to mention that prograde subduction metamorphism has a negligible effect on the Th fractionation over La and the extraction of trace elements are controlled mainly by the residual accessory phases during the sediment melting (Spandler et al., 2003; Tommassini et al, 2011).


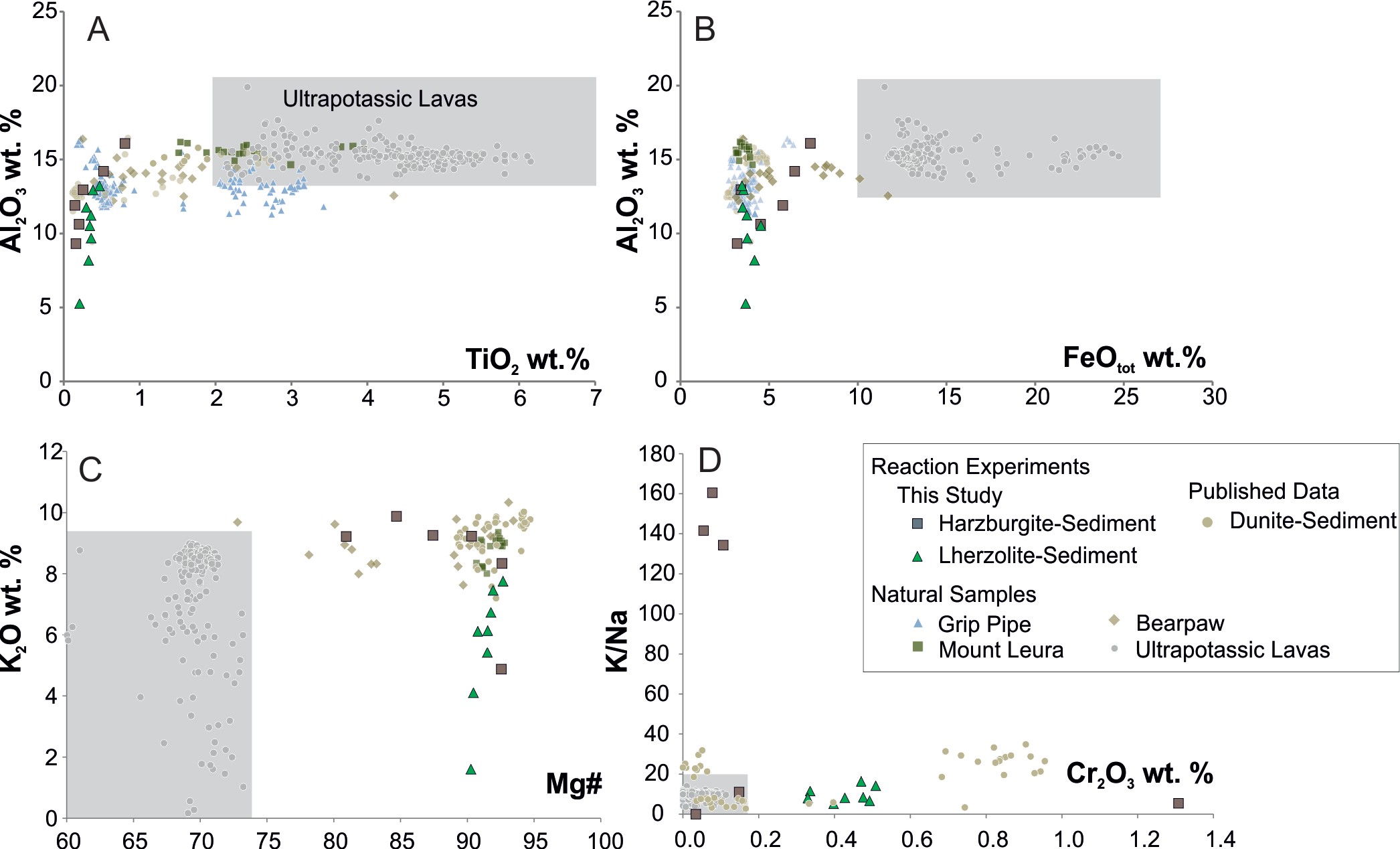


Figure 7: Comparison of the phlogopite from our reaction experiments with others from previous experiments and natural xenolith samples on a) TiO_2_ wt.% vs. Al_2_O_3_ wt.% ,b) FeO wt.% vs. Al_2_O_3_ wt.%, c) Mg# vs. K_2_O wt.% and d) Cr_2_O_3_ wt.% vs. K/Na. Phlogopite in various xenoliths (Mount Leura: phlogopite and phlogopite-amphibole harzburgite (Bonadiman et al. 2021), Grip pipe: clinopyroxene-phlogopite xenolith and phlogopite garnet peridotite (Kargin et al. 2019) , Bearpaw: mica dunite, mica wehrlite, mica clinopyroxenite, mica websterite, glimmerite (Downes et al. 2004), ultrapotassic lavas (Pontide Arc; Gülmez et al. 2015) and sediment- dunite reaction experiments [(Förster](#_bookmark14) et al. 2019).

# Mass Balance and Iron-loss

The complexity of the experimental procedure coupled with the dynamic metasomatism process requires that we should first focus on the smaller components of the total system before we can understand the whole. For this reason, we have performed mass balance calculations reducing the amounts of the products and reactants to metasomes (products) and mantle/melt. For the reaction interface, we can write the reaction as follows:

*harzburgite* + *melt*_1_ + *melt*_2_ = *metasome*

which can be further elaborated:

*harzburgite* + *melt*_1_ + *melt*_2_ = *ol* + *sp* + *opx*_m_ + *cpx*_m_ + *opx*_r_ + *cpx*_rz_ + *phl* + *dol*_liquid_

where Melt_1_ is silicic and Melt_2_ is carbonatitic in composition. Ol: olivine, opx: orthopyroxene, sp: spinel, opx_m_: meta- somatic orthopyroxene, cpx_m_: metasomatic clinopyroxene, opx_r_: reaction orthopyroxene, cpx_rz_: rection clinopyroxene in mantle-sediment interface, phl: phlogopite and dol_liquid_: dolomitic glass in mantle portion.

The starting harzburgite is represented by the mineral assemblage of olivine 0.7 + orthopyroxene 0.25 + spinel

0.02 + clinopyroxene 0.03. As Fiji ImageJ was unable to differentiate olivine and orthopyroxene, we have retained their original proportions in harzburgite and excluded the void and crust. By extrapolating the area fractions for mineral proportions, we calculated the mass balance for the above reaction (Table 4). It is likely that for the designated phases and proportions are not suitable to maintain TiO_2_, Al_2_O_3_ and Na_2_O balanced within the system. The 6- and 2-fold increases in the TiO_2_and Al_2_O_3_ respectively from 0.01 to 0.06 and from 0.94 to 2.12 might have resulted from cumulative analytical errors, considering that the initial bulk composition was not enriched in these element oxides. The metasomatic clinopyroxene in our experiments is the main repository for Na_2_O, on which we have a number of measurements. The core to rim analysis reveals oscillations for most of the element oxide contents, whereas FeO, MgO and CaO were found to have changed slightly or not at all (Fig. [9).](#_bookmark8) The texturally homogeneous metasomatic clinopyroxene grains located at the reaction zone display embayment relationships with orthopyroxene. These grains are also in contact with metasomatic growths. These relationships might provide evidence that the equilibrium has not yet been reached. Moreover, clinopyroxene is one of the major phases along with orthopyroxene that is consumed to form metasomes.


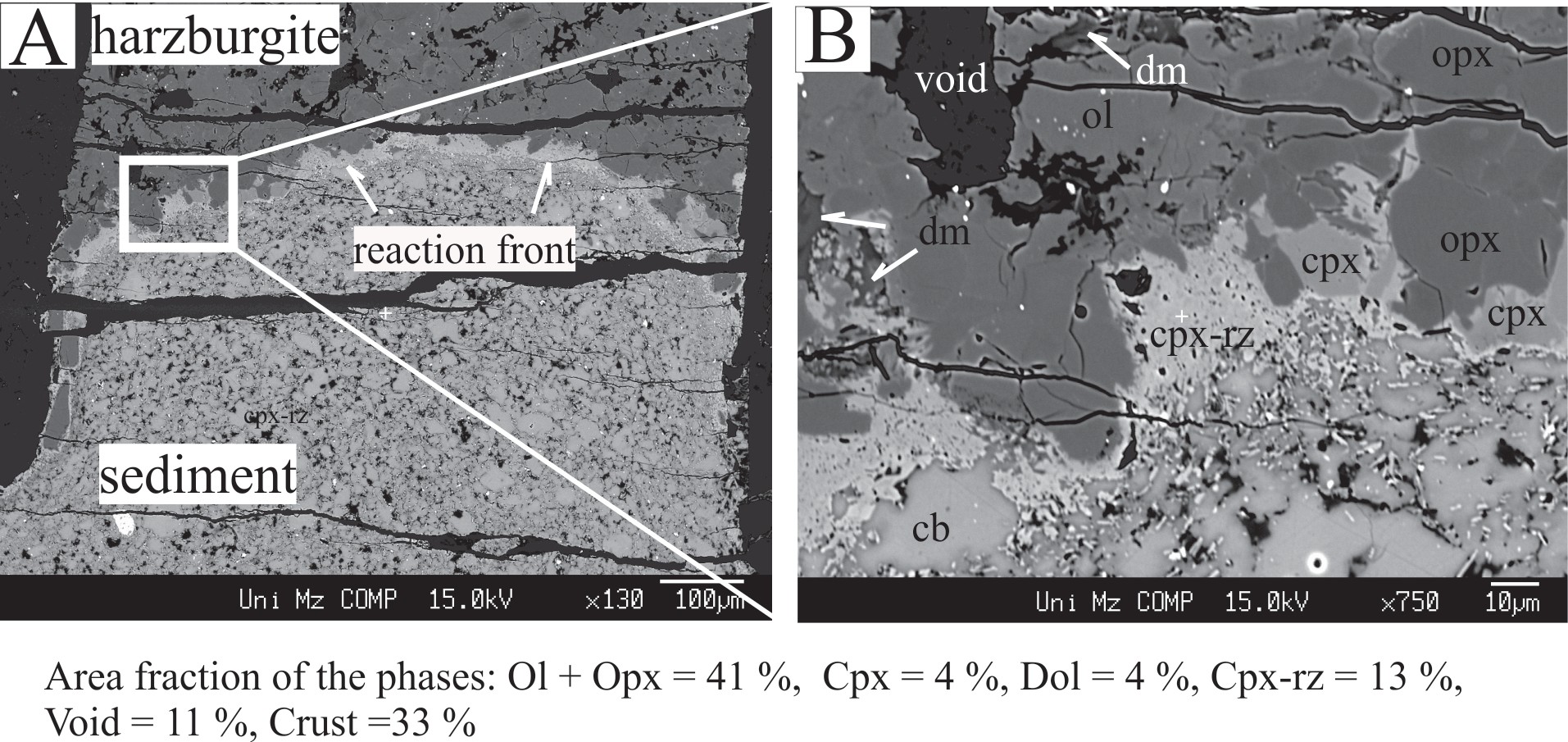


Figure 8: a) The general view of the reaction front in the experiment E10, b) larger scale view of the reaction front showing the phases. The area fraction of the phases was calculated using Fiji ImageJ. Ol: olivine, opx: orthopyroxene, cpx_m_: metasomatic clinopyroxene, cpx_rz_: reaction clinopyroxene, dol: dolomite.


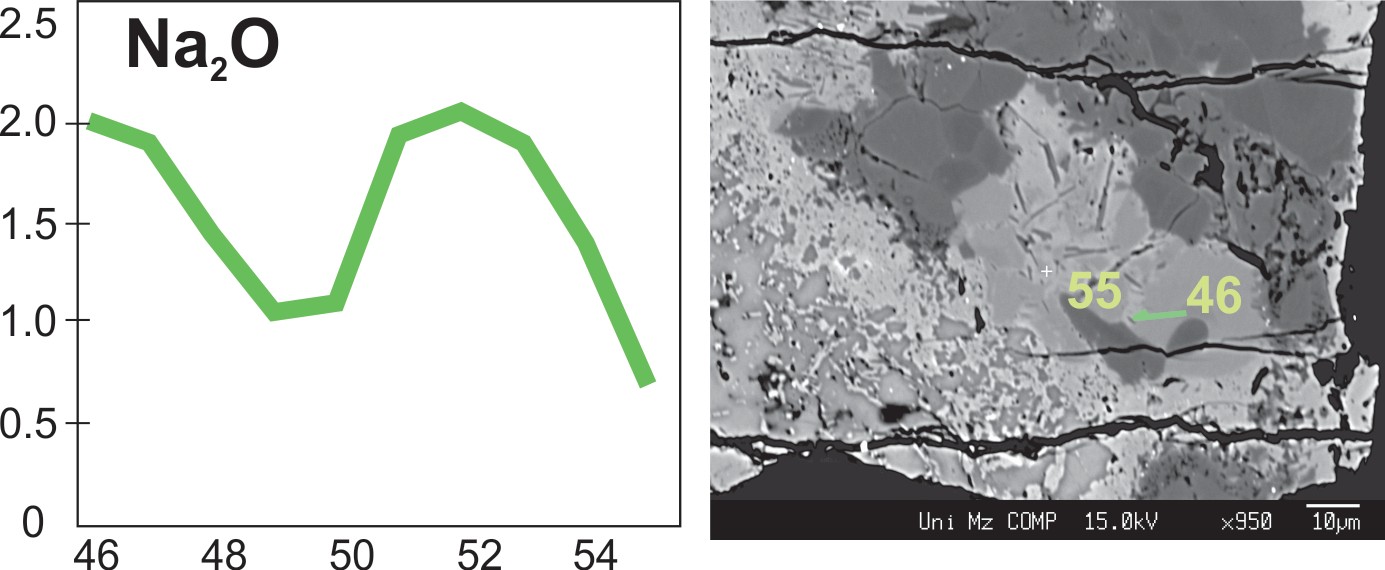


Figure 9: a) The change Na_2_O from core to rim on texturally homogeneous metasomatic clinopyroxene grain in harzburgite-sediment reaction experiment b) The clinopyroxene is in contact with a metasomatic growth consisting of second generation clinopyroxene and phlogopite needles, along its border marked by the 55. measurement point.

10

Table 4: Mass balance calculations for the harzburgite-sediment reaction experiment (Hzb: Harzburgite, Carb. Melt: Carbonatitic Melt, sil. Melt: Silicic Melt, Bulk Comp: Bulk Composition, Bulk Comp*: Bulk composition normalized to !00 wt.%, Ol: Olivine, Sp: Spinel, Opx: Orthopyroxene, Cpx_m_: Metasomatic Clinopyroxene, Opx_r_: Reaction Orthopyroxene, Cpx_rz_: Reaction Zone Clinopyroxene, Ph: Phlogopite, Dl: Dolomite, Calc. Bulk Comp: Calculated Bulk Composition, Calc. Bulk Comp*: Calculated Bulk Composition normalized to !00 wt.%.

| Harzburgite + Melt _1_+ Melt _2_ = Olivine + Spinel + Orthopyroxene_(m)_ + Clinopyroxene_(m)_+ Orthopyroxene_(r)_+ Clinopyroxene_(rz)_ + Phlogopite + Dolomite | | | | | | | | | | | | | | | | | | | | | | |
| --- | --- | --- | --- | --- | --- | --- | --- | --- | --- | --- | --- | --- | --- | --- | --- | --- | --- | --- | --- | --- | --- | --- |
|  |  |  |  |  |  |  |  |  |  |  |  |  |  |  |  |  |  |  |  |  |  |  |
|  |  | Reactants | | |  | Mantle |  | Mantle |  | Phases | | | | | | | |  | Cal. | Cal. |  |  |
|  |  | Hzb | Carb. | Sil. |  | + |  | + |  | Ol | Sp | Opx | Cpxm | Opxr | Cpxrz | Ph | Dl |  | Bulk | Bulk | Deviation | (Mantle+ Melts)- |
|  |  |  | Melt | Melt |  | Melts |  | Melts* |  |  |  |  |  |  |  |  |  |  | Comp | Comp* |  | Calc. Bulk Comp. |
| SiO_2_ |  | 44.43 | 1.40 | 70.25 |  | 43.14 |  | 43.70 |  | 40.83 | 1.88 | 56.98 | 54.67 | 58.21 | 52.24 | 43.25 | 0.09 |  | 44.20 | 44.07 | 2.64 | -1.13 |
| TiO_2_ |  | 0.01 | 0.01 | 0.07 |  | 0.01 |  | 0.01 |  | 0.01 | 0.31 | 0.06 | 0.15 | 0.02 | 0.21 | 0.26 | 0.00 |  | 0.08 | 0.08 | 779.64 | -0.07 |
| Al_2_O_3_ |  | 0.65 | 0.47 | 15.19 |  | 1.36 |  | 1.38 |  | 0.19 | 30.09 | 2.15 | 3.98 | 0.12 | 5.23 | 12.96 | 0.01 |  | 2.13 | 2.12 | 125.99 | -1.18 |
| FeO |  | 7.87 | 5.96 | 0.06 |  | 7.36 |  | 7.46 |  | 8.96 | 13.77 | 4.96 | 3.23 | 6.61 | 3.74 | 3.41 | 8.10 |  | 6.45 | 6.43 | 16.50 | 1.27 |
| MnO |  | 0.11 | 0.23 | 0.00 |  | 0.11 |  | 0.11 |  | 0.09 | 0.16 | 0.09 | 0.09 | 0.10 | 0.07 | 0.00 | 0.12 |  | 0.08 | 0.08 | 30.36 | 0.04 |
| MgO |  | 44.47 | 3.93 | 0.01 |  | 39.81 |  | 40.33 |  | 50.30 | 17.94 | 32.84 | 19.60 | 35.64 | 15.83 | 23.97 | 66.29 |  | 37.84 | 37.72 | 9.54 | 3.98 |
| CaO |  | 0.88 | 86.84 | 0.45 |  | 6.02 |  | 6.10 |  | 0.16 | 0.20 | 2.29 | 14.70 | 0.32 | 20.35 | 2.55 | 24.61 |  | 7.01 | 6.99 | 14.28 | -0.87 |
| Na_2_O |  | 0.14 | 0.06 | 0.93 |  | 0.17 |  | 0.17 |  | 0.02 | 0.04 | 0.29 | 1.76 | 0.00 | 0.64 | 0.50 | 0.04 |  | 0.27 | 0.27 | 77.53 | -0.12 |
| K_2_O |  | 0.01 | 0.36 | 12.64 |  | 0.66 |  | 0.67 |  | 0.00 | 0.01 | 0.01 | 0.02 | 0.00 | 0.05 | 8.33 | 0.01 |  | 0.31 | 0.31 | 7.64 | -0.02 |
| Total |  | 98.55 | 100.00 | 100.00 |  | 98.71 |  | 100.00 |  | 101.55 | 99.69 | 100.59 | 100.34 | 101.62 | 99.73 | 97.41 | 100.00 |  | 100.31 | 100.00 | 0.00 | 0.00 |
| Proportions | | |  |  |  |  |  |  |  |  |  |  |  |  |  |  |  |  |  |  |  |  |
| % |  | 0.92 | 0.06 | 0.02 |  |  |  |  |  | 0.39 | 0.01 | 0.24 | 0.05 | 0.01 | 0.17 | 0.06 | 0.08 |  |  | R^2^ = | 0.9999 |  |

# References

Andersen, T. & Neumann, E. R. Fluid inclusions in mantle xenoliths. *Lithos* **55(1-4)**, 301-320 (2001).

Arai, S. (1986). K/Na variation in phlogopite and amphibole of upper mantle peridotites due to fractionation of the metasomatizing fluids. The Journal of Geology, 94(3):436–444, 1986.

Avanzinelli, R., Elliott, T., Tommasini, S. & Conticelli, S. Constraints on the genesis of potassium-rich Italian volcanic rocks from U/Th disequilibrium. J. Petrology 49, 195–223 (2008).

Bonadiman, C., Brombin, V., Andreozzi, G.B., Benna, P., Coltorti, M., Curetti, N., Faccini, B., Merli, M., Pelorosso, B., Stagno, V. (2021) Phlogopite-pargasite coexistence in an oxygen reduced spinel-peridotite ambient. *Scientific Reports*, 11(1):1–17.

Chen, C., Förster, M. W., Foley, S. F., & Liu, Y. (2021). Massive carbon storage in convergent margins initiated by subduction of limestone. Nature Communications, 12(1), 4463.

Conticelli, S., Guarnieri, L., Farinelli, A., Mattei, M., Avanzinelli, R., Bianchini, G., ... & Venturelli, G. (2009). Trace elements and Sr–Nd–Pb isotopes of K-rich, shoshonitic, and calc-alkaline magmatism of the Western Mediterranean Region: genesis of ultrapotassic to calc-alkaline magmatic associations in a post-collisional geodynamic setting. Lithos, 107(1-2), 68-92.

Downes, H., Macdonald, R. A. Y., Upton, B. G., Cox, K. G., Bodinier, J. L., Mason, P. R., ... & Hearn Jr, B. C. (2004). Ultramafic xenoliths from the Bearpaw Mountains, Montana, USA: Evidence for multiple metasomatic events in the lithospheric mantle beneath the Wyoming craton. Journal of Petrology, 45(8), 1631-1662.

Ezad, I. S., Shcheka, S. S., Buhre, S., Buhre, A., Gorojovsky, L. R., Shea, J. J., ... & Foley, S. F. Rapid quench piston cylinder apparatus: An improved design for the recovery of volatile-rich geological glasses from experiments at 0.5–2.5 GPa. *Review of Scientific Instruments* **94(5)**, (2023).

Förster, M. W., Prelević, D., Buhre, S., Mertz-Kraus, R., & Foley, S. F. (2019). An experimental study of the role of partial melts of sediments versus mantle melts in the sources of potassic magmatism. Journal of Asian Earth Sciences, 177, 76-88.

Förster, M. W., Bussweiler, Y., Prelević, D., Daczko, N. R., Buhre, S., Mertz-Kraus, R., & Foley, S. F. (2021). Sediment-peridotite reaction controls fore-arc metasomatism and arc magma geochemical signatures. Geosciences, 11(9), 372.

Gülmez, F., Genç, Ş. C., Prelević, D., Tüysüz, O., Karacik, Z., Roden, M. F., & Billor, Z. (2016). Ultrapotassic volcanism from the waning stage of the Neotethyan subduction: a key study from the Izmir–Ankara–Erzincan Suture Belt, Central Northern Turkey. Journal of Petrology, 57(3), 561-593.

Green, D. H., & Wallace, M. E. (1988). Mantle metasomatism by ephemeral carbonatite melts. *Nature*, *336*(6198), 459-462.

Kargin, A. V., Sazonova, L. V., Nosova, A. A., Lebedeva, N. M., Kostitsyn, Y. A., Kovalchuk, E. V., ... & Tikhomirova, Y. S. (2019). Phlogopite in mantle xenoliths and kimberlite from the Grib pipe, Arkhangelsk province, Russia: evidence for multi-stage mantle metasomatism and origin of phlogopite in kimberlite. Geoscience Frontiers, 10(5), 1941-1959.

Kepezhinskas, P.K., Defant, M.J & Drummond, M.S. Na metasomatism in the island- arc mantle by slab melt—peridotite interaction: evidence from mantle xenoliths in the north kamchatka arc. *Journal of Petrology*, 36(6):1505–1527, 1995.

Mallik, A., Dasgupta, R., Tsuno, K., & Nelson, J. (2016). Effects of water, depth and temperature on partial melting of mantle-wedge fluxed by hydrous sediment-melt in subduction zones. Geochimica et cosmochimica acta, 195, 226-243.

Matveev, S., O’neill, H. S. C., Ballhaus, C., Taylor, W. R., & Green, D. H. (2001). Effect of silica activity on OH− IR spectra of olivine: implications for low-a SiO2 mantle metasomatism. Journal of Petrology, 42(4), 721-729.

Menzies, M. A., Rogers, N., Tindle, A., & Hawkesworth, C. J. (1987). Metasomatic and enrichment processes in lithospheric peridotites, an effect of asthenosphere-lithosphere interaction. Mantle metasomatism, 313-361.

Poli, S. (2015). Carbon mobilized at shallow depths in subduction zones by carbonatitic liquids. *Nature Geoscience*, 8(8):633–636.

Rapp, R. P., Shimizu, N., Norman, M. & Applegate, G. Reaction between slab-derived melts and peridotite in the mantle wedge: experimental constraints at 3.8 Gpa. chemical Geol. 160, 335–356 (1999)

Sekine, T., & Wyllie, P. J. (1983). Experimental simulation of mantle hybridization in subduction zones. The Journal of Geology, 91(5), 511-528.

Spandler, C., Hermann, J., Arculus, R., & Mavrogenes, J. (2003). Redistribution of trace elements during prograde metamorphism from lawsonite blueschist to eclogite facies; implications for deep subduction-zone processes. Contributions to Mineralogy and Petrology, 146, 205-222.

Thomsen, T. B., & Schmidt, M. W. (2008). Melting of carbonated pelites at 2.5–5.0 GPa, silicate–carbonatite liquid immiscibility, and potassium–carbon metasomatism of the mantle. Earth and Planetary Science Letters, 267(1-2), 17-31.

Tommasini, S., Avanzinelli, R., & Conticelli, S. (2011). The Th/La and Sm/La conundrum of the Tethyan realm lamproites. Earth and Planetary Science Letters, 301(3-4), 469-478.

Wyllie, P. J., & Sekine, T. (1982). The formation of mantle phlogopite in subduction zone hybridization. Contributions to Mineralogy and Petrology, 79, 375-380.
